# Optical bio/chemical sensors for vitamin B_12_ analysis in food and pharmaceuticals: state of the art, challenges, and future outlooks

**DOI:** 10.3762/bjnano.16.153

**Published:** 2025-12-05

**Authors:** Seyed Mohammad Taghi Gharibzahedi, Zeynep Altintas

**Affiliations:** 1 Division of Bioinspired Materials and Biosensor Technologies, Institute of Materials Science, Faculty of Engineering, Kiel University, 24143, Kiel, Germany; 2 Kiel Nano, Surface and Interface Science (KiNSIS), Kiel University, 24118, Kiel, Germany

**Keywords:** carbon dots, cobalamin, energy transfer, fluorescence sensor, molecularly imprinted polymers (MIPs), nanobiosensor

## Abstract

Vitamin B_12_ (VB_12_) is an essential Co^2+^-containing nutrient for neurological function, DNA synthesis, and red blood cell formation. Accurate and efficient VB_12_ quantification in food and pharmaceutical products is crucial due to its animal-derived dietary sources and the significant health implications of VB_12_ deficiency. Traditional methods for VB_12_ analysis, such as high-performance liquid chromatography and enzyme-linked immunosorbent assay, are often troublesome and time-consuming, and require high-tech laboratory setups. The current overview highlights the latest optical biosensing platforms in detecting Co^2+^ ions and VB_12_ using RNA aptamer–gold nanoparticles colorimetric sensors, surface plasmon resonance sensors, chemiluminescence and electrochemiluminescence biosensors, and fluorescence biosensors (i.e., chemosensors, nanoclusters/nanoparticles-based sensors, and carbon dot (CD)- and quantum dot (QD)-based sensors). The advent of optical biosensing technologies has resulted in a new era for VB_12_ analysis, characterized by the development of innovative CD- and QD-based sensors. These nanomaterials offer several advantages over conventional methods, including enhanced sensitivity, specificity, rapid detection, and the ability for real-time analysis. CD- and QD-based biosensors with excellent optical properties such as photoluminescence enable the detection of VB_12_ at negligible concentrations and in real-world samples with complex matrices. Furthermore, integrating these biosensors into cellular bioimaging and the potential for non-invasive in vitro and in vivo analysis demonstrate their versatility and applicability across a broad spectrum of biomedical research, diagnostics, and nutrient analysis.

## Introduction

Micronutrients including vitamins and minerals play key roles in modulating body growth, preventing a wide range of diseases and disorders, and maintaining general health and wellness [1–2]. Apart from vitamin D, which the body can synthesize under sunlight exposure, all other micronutrients must be obtained via dietary intake [3]. Vitamins are classified into two distinct categories, namely, water-soluble (e.g., vitamin C and vitamin B group) and fat-soluble (e.g., vitamins A, D, E, and K). Vitamin B_12_ (VB_12_) is among B-group vitamins and cannot be absorbed through plant sources. This vitamin should be provided by consuming animal-derived products such as milk and dairy products, meat and meat products (e.g., liver, poultry, beef, pork, and ham), eggs, fish (e.g., tuna, trout, sardine, and salmon), and shellfish [2]. Recently, the presence of this vitamin in some plant sources such as microalgae (e.g., *Spirulina* and *Chlorella*) and mushrooms (e.g., shiitake, maitake, black trumpet, and golden chanterelle) species and Asian fermented soy products (e.g., tempeh and miso) has been reported [2,4–5]. Since these plant sources and their derived products cannot provide adequate amounts of active VB_12_ for the human body, the supplementation of plant products such as breakfast cereals and nondairy milk is considered a possible dietary strategy for preventing its deficiency among vegetarians and vegans [2,6–7]. The deficiency of VB_12_ can lead to several health issues such as pernicious anemia (PA), anemia, fatigue, nausea, and weight loss [2,8]. In contrast, excessive intake of VB_12_ may contribute to conditions like liver disease, neurotoxicity, kidney failure, or myeloproliferative disorders [2,9–10]. Consequently, monitoring the levels of VB_12_ in foods and pharmaceuticals is crucial for health management and disease prevention.

**Figure 1:**
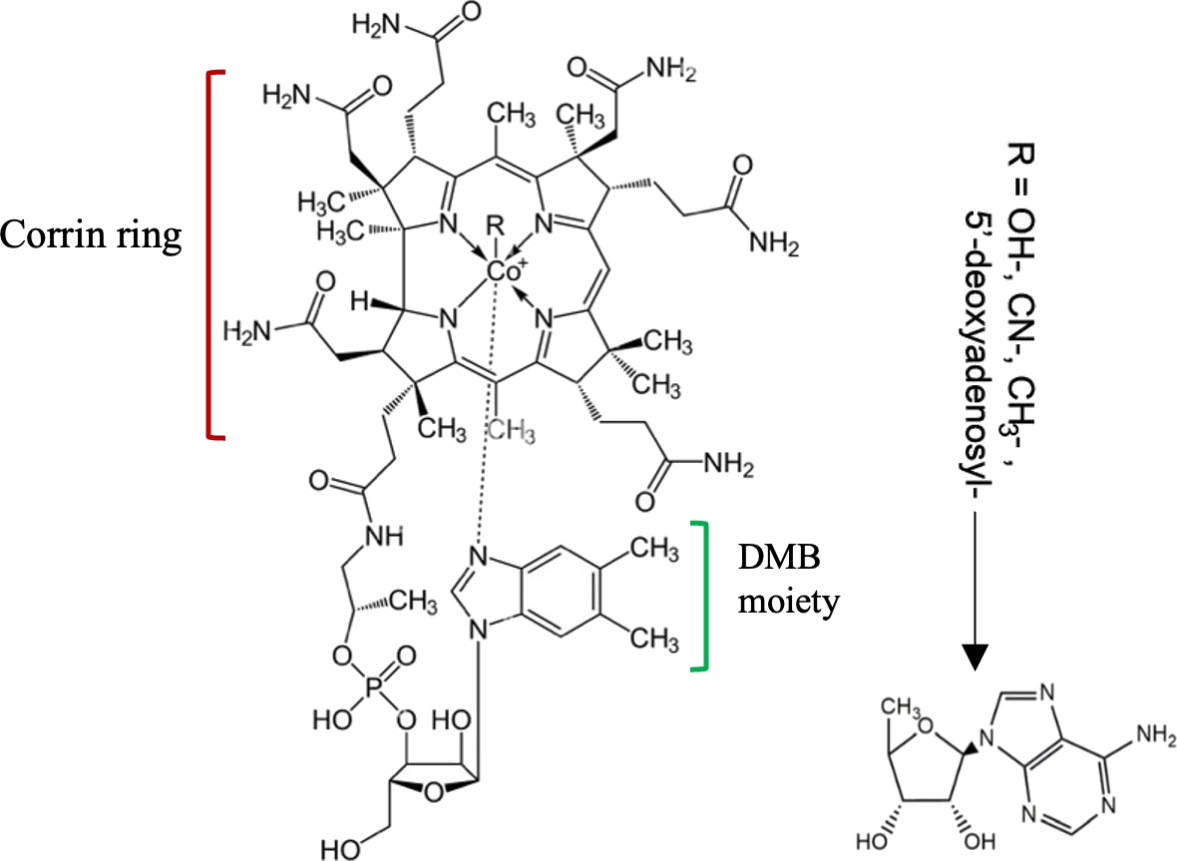
The chemical structure of VB_12_ (C_63_H_88_CoN_14_O_14_P; 1355.388 g/mol). Figure 1 was adapted from [2] (© 2023 S. M. T. Gharibzahedi et al., published by MDPI, distributed under the terms of the Creative Commons Attribution 4.0 International License, https://creativecommons.org/licenses/by/4.0).

VB_12_ is also known as cobalamin due to the presence of a central cobalt ion (Co^2+^) within the structure of its modified tetrapyrrole ring (Figure 1). A unique feature of the tetrapyrrole-derived ring in VB_12_ is that it has experienced a process called ring contraction. In this change, one of the carbon atoms that usually links the four pyrrole rings together is removed. This alteration results in a tighter and unevenly shaped large ring (corrin), making it different from the broader and more symmetrical rings found in heme and chlorophyll [2,11]. The structure of VB_12_ also includes a nucleotide loop that contains a unique base known as dimethylbenzimidazole (DMB). This nucleotide loop is attached to one of the propionate side chains of the corrin ring via an aminopropanol linker and stretches out below the corrin ring’s plane. This particular arrangement allows the DMB base to act as a secondary ligand for the central cobalt ion (Co^2+^), playing a crucial role in the molecule’s structural integrity and biological activity. Within the structure of VB_12_, Co^2+^ is capable of binding to an upper ligand, which can vary among different biochemical forms (–R, Figure 1), including hydroxy (OH–, OHCbl), cyanide (CN–, CNCbl), methyl (CH_3_–, MeCbl), and adenosyl (AdoCbl) [2]. Nonetheless, CNCbl is the main form of this vitamin, as cyanide is commonly used to extract and purify the vitamin during the isolation process [2,12].

Quality control measures in the industry include the analysis of VB_12_ concentrations in serum extracted from blood plasma, pharmaceutical products such as tablets and injections, and fermentation-derived products [13]. The most common techniques to determine the VB_12_ content in different food and pharma formulations include high-performance liquid chromatography (HPLC) [14], HPLC coupled with inductively coupled plasma-mass spectrometry (ICP-MS) [15], and a diode array detector [16], atomic absorption spectroscopy [17], surface-enhanced Raman spectroscopy [18–19], capillary electrophoresis [20], chemiluminescence [21–24], enzyme-linked immunosorbent assay (ELISA) [25–26], and electrochemical assays [2,27]. However, these methods involve complex procedures with long preparation times, poor selectivity, expensive equipment, and relatively costly or environmentally harmful reagents, which restrict their practical applications [2,13,28]. Consequently, there is a growing need to develop simple, rapid analytical systems such as biosensors to tackle these challenges by highly selective and sensitive detection of VB_12_ without requiring specialized skills.

Nowadays, electrochemical and optical biosensing platforms are one of the best approaches to detect VB_12_. Various electrochemical methods have been employed for the effective detection of VB_12_, focusing on its electroactive nature and the redox chemistry of the cobalt atom, primarily utilizing the Co^3+^/Co^2+^ and Co^2+^/Co^+^ redox reactions [2,27]. Although Antherjanam et al. reviewed different electrochemical sensing strategies for VB_12_ [27], to the best of our knowledge, the utilization of optical sensing platforms for detecting VB_12_ has not been yet reviewed. Optical sensing involves generating an optical signal as the output, utilizing the optical properties of the signal such as fluorescence, absorbance, refractive index, and Raman scattering for quantification and evaluation. The most prevalent types of optical sensing include colorimetric, plasmonic, fluorescence, and spectrophotometric methods due to their ease of use, affordability, and enhanced performance [29–30]. The current study critically reviews the newest findings on the mechanisms and designing principles of different optical sensing systems for sensitive and selective detection of VB_12_ in various media and real-world samples. Present limitations and emerging trends in a wide range of optical sensing systems of VB_12_ in analyzing food and pharmaceutical products are also highlighted.

## Review

### Nutritional requirements of vitamin B_12_

The recommended daily intakes (RDIs) of VB_12_ in the UK and the US are 2.4 µg and 1.5 µg, respectively, which should be obtained by consuming animal-derived products, fortified foods, and vitamin supplements [2,31–34]. However, a higher dietary intake of VB_12_ for pregnancy and lactation is recommended. The RDIs of VB_12_ during lactation in the UK and the US are 2.0 and 2.8 µg, respectively. However, the RDI of VB_12_ for adults in the European Union is 4.0 µg [32–33]. The bioavailability of VB_12_ in food sources for healthy adults with typical absorption efficiency is roughly estimated to be 50%. In contrast, crystalline VB_12_ incorporated into supplements and fortified foods shows an absorption rate ranging from 55% to 74%. Hence, it is important to note that the absorption rates vary greatly depending on the specific food items; for instance, egg products, fish, and lean meat have an absorption rate of 24% to 36% (VB_12_ dose 0.30–0.94 µg), 42% (dose 1.95–2.18 µg), and 65% (dose 0.95 µg), respectively [34–35]. This water-soluble micronutrient is essential for improving brain and nervous system functions, blood cell development, bone health improvement, energy and DNA production, fertility and embryo development, control of neurological symptoms (e.g., stress, depression, dementia, and visual disturbances), and fatigue reduction [2,36]. VB_12_ deficiency represents a significant global public health concern, impacting approx. 6% of the global population and 1.6% to 10% of European communities [37]. The different demographic data showed that the most vulnerable groups to VB_12_ deficiency comprise geriatric populations, vegetarians and vegans, pregnant women, and breastfeeding infants of VB_12_-deficient mothers, particularly in developing countries [10,38–40]. VB_12_ has a pivotal role in the development of the fetal and neonatal brain. Therefore, mothers with vegetarian and vegan diets should significantly increase the intake rate of this vitamin during pregnancy and lactation as VB_12_ deficiency can cause some fully reversible damage to newborns’ brain and nervous system health [41–42]. A recent study revealed that VB_12_ deficiency should be considered one of the most important parameters in infants with hypotonia or neurodevelopmental retardation accompanied by thinning of the corpus callosum, cortical atrophy, and retardation in myelination [43]. The inadequate intake of VB_12_ from daily diets becomes clinically apparent after several years because of the substantial hepatic storage capacity (1–5 mg) and minimal losses via enterohepatic circulation [44]. Yet, this nutritional deficiency can quickly appear with VB_12_ malabsorption in patients with PA, celiac disease, inflammatory bowel disease, Whipple’s disease, food-bound VB_12_ malabsorption, chronic alcoholism, patients taking antidiabetic medications (e.g., metformin) and bile acid sequestrants (e.g., cholestyramine), and patients with inherited disorders such as deficiency of the non-glycosylated protein of transcobalamin II (TC-II) [2,10,45–48].

### Pathways of vitamin B_12_ absorption and metabolism

**Figure 2:**
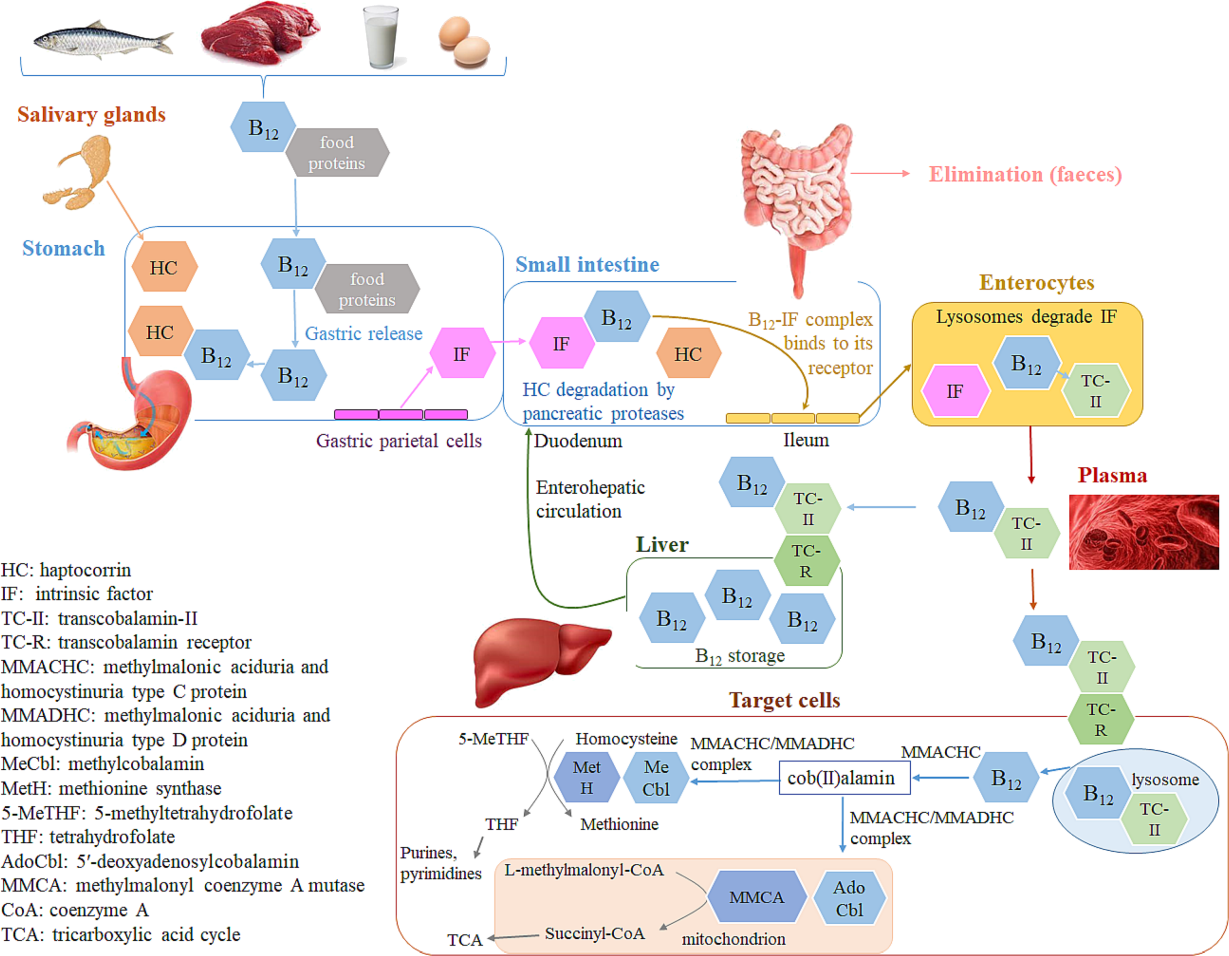
Graphical illustration of the ADME (absorption, distribution, metabolism, excretion) pathways for VB_12_, including its enterohepatic recycling, as well as the mechanisms regulating its cellular absorption and physiological roles. Figure 2 was reproduced from [47] (© 2022 Ž. Temova Rakuša et al., published by MDPI, distributed under the terms of the Creative Commons Attribution 4.0 International License, https://creativecommons.org/licenses/by/4.0).

Two principal mechanisms exist for the absorption of VB_12_ (Figure 2), namely, passive diffusion and active transport [34,44]. In passive diffusion, a minor fraction (1–2%) of oral VB_12_ dose can be directly absorbed via the intestinal mucosa and the gastrointestinal tract surface without the need for the intrinsic factor (IF). If the active transport of VB_12_ is impossible, a high dose of oral VB_12_ (like 1.0 mg daily) is essential for ensuring an “adequate” intake of this vitamin in the body [34,49]. Accordingly, this IF-independent pathway is less efficient and becomes more significant in individuals with a deficiency or absence of IF, or when the capacity of the IF system is exceeded [50]. In the active transport pathway, the absorption of VB_12_ through receptors commences following its liberation from the dietary source. Food proteins act as carriers for dietary VB_12_. The acidic conditions within the gastric lumen enable the liberation of this vitamin from food matrices, initiating its active absorption into the body. A glycoprotein known as haptocorrin (HC; transcobalamin-I or R-binder), secreted by salivary and esophageal glands, binds to the released VB_12_ and shields it against the acidic environment of the stomach [47,51–52]. Nevertheless, proteases present in the duodenum are capable of breaking down the VB_12_–HC complex under alkaline conditions, allowing the liberated VB_12_ to easily link to the IF that is secreted from the stomach’s parietal cells [12,44,47–48]. The VB_12_–IF complex moves to the distal ileum, the primary location for its absorption. Here, it attaches to a specific receptor (Cubam) on the ileal enterocyte membrane and is internalized into the enterocytes via endocytosis. Meanwhile, lysosomes break down IF, freeing VB_12_. This allows VB_12_ to bind to TC-II (HoloTC), facilitating its transport in the bloodstream and delivery to target cells [47,53–54].

The VB_12_–TC-II complex is absorbed by cells through receptor-specific endocytosis. Once inside, it undergoes degradation in the lysosomes, resulting in the release of VB_12_. This vitamin inside the target cell is transformed into its two active coenzyme forms (AdoCbl and MeCbl), via a complicated intracellular process involving various chaperone proteins and transporters, regardless of its form when ingested [34,47,50,55]. Acting as the main chaperone, the methylmalonic aciduria and homocystinuria type-C protein (MMACHC) captures VB_12_ exiting the lysosomes in a distinct base-off conformation. In this process, the protein replaces the 5,6-dimethylbenzimidazole ligand of VB_12_ with one of its histidine residues. Furthermore, MMACHC plays a pivotal role in converting all variants of VB_12_ into the cob(II)alamin intermediate. This crucial step includes the decyanation of CNCbl and the dealkylation of alkylcobalamins (alkylCbls). These biochemical transformations are contingent upon the enzymatic action of glutathione S-transferase [47,56–57]. The subsequent transformation of cob(II)alamin into AdoCbl and MeCbl is facilitated by a complex of enzymes (i.e., MMACHC, methylmalonic aciduria and homocystinuria type-D protein (MMADHC), and methionine synthase reductase). The physiological functions of AdoCbl and MeCbl are activated when they bind to their respective target enzymes like methionine synthase (MetH) and methylmalonyl-CoA mutase (MCM) [58–59]. Accordingly, MeCbl emerges as the prevalent form of VB_12_ in plasma, while AdoCbl is dominant across all tissues [47,60]. MeCbl plays a crucial role in the remethylation of homocysteine (Hcy) to methionine, a process catalyzed by methionine synthase. In this reaction, 5-methyltetrahydrofolate (5-MTHF) donates the methyl group and is thereafter transformed into tetrahydrofolate. Methionine is then converted into S-adenosylmethionine (SAM), which serves as a vital methyl donor for the methylation of proteins, phospholipids, neurotransmitters, RNA, and DNA. In contrast, Ado-Cbl acts as a cofactor for MCM, facilitating the transformation of methylmalonyl-CoA into succinyl-CoA, with methylmalonyl-CoA being a byproduct of propionate metabolism. In the majority of mammals, propionate is generated through the catabolism of specific amino acids (e.g., Ile, Met, Thr, Thy, and Val), cholesterol, and the β-oxidation of odd-chain fatty acids [34]. This function of Ado-Cbl in the body supports energy production by facilitating the conversion of methylmalonyl-CoA to succinyl-CoA, a key step in the Krebs cycle, which is central to cellular energy generation [61]. The metabolic pathway of VB_12_ in the body concludes with its primary excretion via bile. The liver then reabsorbs and keeps the excreted VB_12_, finishing its cycle in the body. Excess amounts of this vitamin are also expelled from the body via urine [2,62].

### Monitoring methods of vitamin B_12_ deficiency

In clinical assessments, the deficiency level of VB_12_ can be measured based on the following biomarkers: (i) high levels of corpuscular volume of erythrocytes, (ii) low serum levels of VB_12_, (iii) high plasma levels of total Hcy and methylmalonic acid (MMA), and (iv) decreased serum levels of VB_12_–TC-II complex [10,32,43,47,63]. Cutoff values for target metabolites from literature reports or standard clinical values (SCVs) are as follows: total plasma VB_12_ (i.e., protein-bound VB_12_ and free VB_12_) < 148 pmol/L or 200 ng/L (SCVs) [64–66], HoloTC < 35 pmol/L [65–67], Hcy > 12.6–13.0 μmol/L [65,67], MMA > 350 nmol/L [68–69], erythrocyte folate < 160 μg/L (SCVs) [65], and creatinine > 97 μmol/L (>1.1 mg/dL) for women as well as >124 μmol/L (>1.4 mg/dL) for men (SCVs) [32,65]. In an intriguing review, Aparicio-Ugarriza et al. highlighted the varying cut-off levels for defining VB_12_ deficiency used in studies from different countries, organized by the VB_12_ concentration threshold [70].

Initially, labs used microbiological tests to determine VB_12_ levels because of their favorable accuracy. These tests did not measure VB_12_ directly. Instead, microbiological analyses examined how certain bacteria consume this water-soluble vitamin, especially *Lactobacillus leichmannii* (ATCC 7830). Meanwhile, radioisotope dilution methods also came into use. But now, automated tests, owing to their higher speed and lower labor intensity, are the top choice for determining VB_12_. Since the early 1990s, assays based on competitive-binding luminescence have been used to measure total VB_12_. They include immunoassays such as electrochemiluminescence, chemiluminescence, enzyme-linked fluorescent, or colorimetric methods [71]. Immunoassays are the most common method to determine the total plasma VB_12_. However, this technique can also produce inaccurately normal readings due to interference from anti-intrinsic factor (IF-Ab) or variables like heterophilic antibodies [72–73]. However, Zhang et al. have recently collected VB_12_ through solid-phase extraction and analyzed it using an LC tandem mass spectrometry (LC-MS/MS) method. They showed that the LC-MS/MS assay can efficiently contribute to distinguishing false-normal VB_12_ results reported by immunoassays [72]. ELISA, as well as electrochemiluminescent and chemiluminescent immunoassays, are usually applied to test HoloTC in clinical labs [26]. Not only does the accuracy of HoloTC tests still depend on the specific method but some rare genetic variations in the TC gene may also cause falsely low HoloTC results, even when no real deficiency exists and other lab markers are normal. HoloTC measurements are usually not affected by high IF-Ab levels. However, HoloTC levels in a few cases (particularly with PA) might misleadingly appear normal [26,71]. Looking back, techniques like paper chromatography, thin-layer chromatography, spectrophotometry, and ELISA were employed to measure MMA concentrations. However, mass spectrometry methods, particularly LC-MS/MS, have gradually gained popularity for their enhanced sensitivity and specificity [26]. Currently, automated enzyme immunoassays, GC-MS, LC-MS, and HPLC with either fluorescent or electrochemical detection are utilized to assess the total HCY level [26,71,74].

### Optical biosensing techniques of vitamin B_12_

#### RNA aptamer-based AuNP colorimetric biosensors

In this sensing system, the recognition material is the RNA aptamer, the indicator material is the gold nanoparticle (AuNP), and the sensing signal is colorimetric. The excellent optical-electronic characteristics of AuNPs have been recently considered in point-of-care (POC) diagnostics for different disorders and diseases. Resonance among the free electrons at the surface of colloidal AuNPs, induced by the interaction with light energy, causes the light to be absorbed in the blue-green and red parts of the visible spectrum. Consequently, the appearance of colors in AuNP suspensions is substantially influenced by their particle size and dispersion speed. 10–20 nm AuNPs with a narrow size distribution tend to absorb light from the blue-green spectrum and reflect red light. In contrast, their aggregation leads to a light absorption shift to longer wavelengths, resulting in reflecting light ranging from pale blue to purple [75–76].

Selvakumar and Thakur developed a colorimetric sensor for detecting VB_12_ using AuNPs and a stable modified RNA aptamer. The RNA aptamer, which attaches to VB_12_, triggers the aggregation of AuNPs, resulting in a color shift from red to purple. The sensor’s effectiveness was confirmed with a limit of detection (LOD) of 0.1 μg/mL and a recovery rate (RR) of 92.0–95.3% for VB_12_, equivalent in accuracy to ultraviolet–visible (UV–vis) spectrometry. Since the sensor’s LOD for VB_12_ was above the recommended dietary allowance level (0.02–0.03 μg/mL in food), they emphasized performing an optimization study to reach a lower acceptable LOD for the regulatory standard [77]. Kumudha et al. characterized the HPLC peak of extracts of VB_12_ obtained from the green microalgae *Chlorella vulgaris* using MS/MS, selected ion recording, and multiple reaction monitoring. They found that MeCbl was the main constituent of the extracted VB_12_ and analyzed by chemiluminescence, AuNPs-based RNA aptamers, and microbiological techniques. The corresponding amounts of MeCbl were 26.84, 28.02, and 29.87 μg/100 g dry weight, respectively. Accordingly, there was no significant difference in the detected amounts of MeCbl among the utilized diagnostic assays [78].

#### Small-molecule-based colorimetric chemosensors for cobalt/VB_12_ detection

In this class, the recognition material is a small-molecule receptor (chemosensor), the indicator material is its intrinsic chromophore or fluorophore scaffold, and the sensing signal is primarily colorimetric, with some examples also producing dual colorimetric/fluorescent outputs.

The absence of Co^2+^ in the body can increase the PA risk, as the central Co^2+^ in the corrin ring of VB_12_ plays a pivotal role in iron metabolism to synthesize hemoglobin. Conversely, excessive absorption of Co^2+^ causes some negative impacts on health, including asthma, reduced cardiac output, and heart and lung diseases [79].

A new colorimetric chemosensor fabricated from a pyridyl moiety and a 2-chloro-*N*-(2-((3-nitro-2-oxo-2*H*-chromen-4-yl)amino)phenyl)acetamide group was developed to detect Co^2+^ ions in water with high selectivity. Here, the small molecule served as the recognition receptor, its coumarin scaffold acted as the indicator chromophore, and the sensing signal was a visible change from colorless to pale violet [80]. When anchored to silica, this sensor could effectively oxidize Co^2+^ to Co^3+^ in almost entirely aqueous environment. Na et al. also showed that this chemosensor had a superb capacity to be an optical solid sensor [80]. Maity and Govindaraju earlier reported a colorimetric coumarin-conjugated thiocarbanohydrazone-based chemosensor, which could selectively detect Co^2+^. In this system, the coumarin–thiocarbanohydrazone acted as the recognition element, the coumarin moiety provided the indicator, and the sensing signal was a colorimetric shift from light-yellow to deep-pink with a minimum LOD of 1.0 μM. Furthermore, this sensor also showed potential for bioimaging *Escherichia coli* cells due to its fluorescence response when exposed to Co^2+^ [81].

A new fluorescent–colorimetric chemosensor was designed based on 1,8-bis{2-{*N*-[2′-(8′-hydroxy-9,10-anthraquinon-1-yloxy) ethyl] benzimidazoliumyl} ethoxy}-9′,10′ anthraquinone hexafluorophosphate to detect Co^2+^, in which the benzimidazolium–anthraquinone scaffold acted as the recognition unit, the anthraquinone moiety as the indicator, and the signals included a visible color change from orange to red together with fluorescence quenching. A hypochromatic shift of about 27 nm and a new absorption peak at 487 nm were found in the absorption spectrum. Utilizing this chemosensor in integrating with a smartphone resulted in an LOD of 0.47 μM for Co^2+^ [82]. A coumarin platform was utilized to fabricate a new ratiometric and colorimetric chemosensor for Co^2+^, where the coumarin scaffold functioned as both the recognition unit and the chromophoric indicator, while the sensing response was expressed through ratiometric spectral variation. Upon interacting with this cation, a significant 44 nm shift in its absorption spectra by altering color from yellow to red was recorded, which could be easily seen with the naked eye. Using both standard and ratiometric absorption spectrometry techniques, the linear range of detecting Co^2+^ was found to be 0–10 μM with an LOD of less than 0.31 μM [83].

An innovative biosensing platform (i.e., a thiosemicarbazide-based Schiff-base chemosensor containing a naphthalene moiety (TSNCS)) has been recently designed for the colorimetric detection of Ni^2+^ and Co^2+^ ions, in which the Schiff-base provided the recognition site, the naphthalene group served as the chromophoric indicator, and the detection relied on a distinct colorimetric response. Upon exposure to these ions within aqueous solutions of acetonitrile, TSNCS visibly shifted from colorless to dark yellow, enabling instant and accurate determination with low LODs for Ni^2+^ (0.0114 μM) and Co^2+^ (0.0168 μM). This study also successfully applied paper strips coated with TSNCS for swift ion detection in water environments. Moreover, it was utilized for both quantifiable and descriptive evaluation of Co^2+^ in commercial VB_12_ and real-water samples [84]. Kim et al. employed a new chelated-type Schiff base for creating a colorimetric chemosensor to detect Co^2+^ (0.66 μM LOD) and Cu^2+^ (0.88 μM LOD) ions in water, where the Schiff-base acted as the recognition moiety, the ligand framework functioned as the indicator, and the response was monitored through a straightforward colorimetric change from colorless to yellow [85]. Another dual chemosensor composed of a fluorophore (quinoline) and a hydrophilic functional group (*N*^1^,*N*^1^-dimethylethane-1,2-diamine) was fabricated to detect Zn^2+^ (0.01 μM LOD) and Co^2+^ (6.89 μM LOD) in both biological systems and aqueous environments, with the diamine serving as the recognition unit, quinoline as the indicator scaffold, and the outputs expressed as fluorescence enhancement for Zn^2+^ and a visible chromatic shift for Co^2+^. An increase in the fluorescence intensity and a color transition from colorless to yellow led to the determination of these cations, respectively [86]. Alhalafi [87] has currently explored that the reaction of 3-amino-2-styrylquinazolin-4(3*H*)-one with phenols in a diazotization-like process produced a range of azo derivatives, 3-(diazenyl)-2-(styryl)quinazolin-4(3*H*)-one for detecting metal ions of Co^2+^ and Fe^2+^, where the azo-quinazolinone acted as the recognition material, the azo chromophore acted as the indicator, and the sensing readout was a distinct spectral absorption response. Specifically, the derived compound of 3-((2-hydroxynaphthalen-1-yl)diazenyl)-2-(styryl)quinazolin-4(3*H*)-one emerged as a rapid tool for the determination of Co^2+^ (λ_max_ = 582 nm) and Fe^2+^ (λ_max_ = 566 nm) in water.

#### Surface plasmon resonance-based biosensors

Surface plasmon resonance (SPR) sensors operate through a simple and effective mechanism in five key steps: (i) An electromagnetic field at the metal–dielectric interface excites coherent electron oscillations in the metal; (ii) this leads to the generation of surface plasmon polaritons (SPPs; i.e., oscillating charge densities), (iii) SPPs produce a decaying electric field that extends into the surrounding medium; (iv) the evanescent field is highly sensitive to changes in the medium’s refractive index; and (v) during resonance, incident light absorption at a specific angle or wavelength results in a signal peak, which shifts with any refractive index changes due to the analyte [88–89].

Gao et al. applied biomolecular interaction analysis based on SPR to assess B-group vitamins such as VB_12_ enriched into infant formulas based on milk, whey protein concentrate, lactose-free milk protein isolate, or partially hydrolyzed nonfat milk, where the recognition was mediated by VB_12_-binding proteins, the SPR sensor chip acted as the indicator surface, and the sensing signal was generated through resonance shifts. To prepare the extraction buffer, a 0.2% sodium cyanide solution was diluted with a phosphate–citrate buffer at pH 4.5. This buffer was then added to an infant formula sample, which was subsequently autoclaved at 121 °C for 25 min. The purpose of this step was to denature the VB_12_ binding protein present in the sample and liberate the vitamin for the subsequent analysis. Low repeatability based on relative standard deviation (RSD < 2%) with a high RR (94.7–109.1%) was reported for all B-group vitamins [90]. This study was in the continuation of works performed by Indyk et al. [91] and Cannon and colleagues [92]. Indyk et al. applied a similar technique to measure VB_12_ in various foods (i.e., dried and fluid milk, infant formula, cereal-based foods, baby food composite, meat, and liver) using a non-labeled inhibition protein-binding assay, in which the recognition relied on protein–vitamin binding, the sensor surface acted as the indicator, and the signal was measured by resonance response. The quantitation range and RR were 0.08–2.40 ng/mL and 89–106%, respectively [91]. Cannon et al. employed SPR to indirectly detect VB_12_ by observing the interactions between this vitamin and its specific binding proteins, with the protein interaction acting as the recognition step, the SPR chip as the indicator, and the resonance change as the output signal. This method achieved an LOD of approximately 1 mg/mL for VB_12_ [92]. In the Association of Official Agricultural Chemists (AOAC) international meeting on June 29, 2011, the committee eventually decided to collaboratively assess VB_12_ by SPR, specifically for infant formula and adult nutritionals through the Biacore Q™ biosensor and Qflex™ Kit (RSD of 1.59–27.8%). They confirmed that the method satisfies the performance requirements set by the stakeholder panel on infant formula and adult nutritionals for infant and pediatric nutritional formulas [93].

SPR sensors were also applied to determine VB_12_ in ten commercial milk powders in less than 6 h, where covalently immobilized VB_12_ on a CM5 chip acted as the recognition element, the chip surface itself served as the indicator platform, and the sensing signal was derived from resonance changes. The vitamin chip was stable, with an RSD of less than 10% over 50 cycles. Also, the LOD for VB_12_ was 0.006 µg/100 g with an RR of 92.1–104.1% [94]. Çimen and Denizli have recently developed a plasmonic nanosensor for the real-time detection of B-group vitamins (i.e., B_2_, B_9_, and B_12_) in infant formula and milk samples using molecular imprinted polymers (MIPs), where the MIP cavities served as the recognition sites, the SPR chip surface as the indicator, and the resonance shift as the sensing signal. For SPR chip surface modification, they initially added allyl groups to the SPR chip surface using allyl mercaptan. To eliminate any unattached allyl mercaptan, the chip underwent washing with distilled water and ethanol, followed by drying under vacuum at 200 mmHg and 25 °C. The determined LOD for vitamins B_2_, B_9_, and B_12_ were 1.6 × 10^−4^, 13.5 × 10^−4^, and 2.5 × 10^−4^ ng/mL, respectively. Not only was a remarkably excellent selectivity, reproducibility, and storage stability for the SPR sensor recorded, but a strong association between the SPR sensor and LC-MS/MS findings was found [95].

Recently, Bareza et al. have reported that graphene nanostructures, which can confine mid-infrared plasmons at the nanoscale, serve as an advanced spectroscopic platform for improved molecular identification. They highlighted graphene’s potential in biosensing owing to its capability to be functionalized with dissimilar biomolecules such as enzymes and DNA. In their study, they presented a quantitative bioassay leveraging mid-IR plasmon resonance in graphene nanostructures to detect VB_12_, where functionalized graphene nanoribbons provided the recognition sites, the nanostructured graphene surface acted as the plasmonic indicator, and the sensing signal was a mid-IR resonance shift. This approach achieved an LOD of 53.5 ng/mL using graphene nanoribbons modified with specific recognition elements [96]. Moreover, Bareza et al. demonstrated the scalability and industrial applicability of this bioassay through the use of large-area nanostructured graphene films, proving the promising future of graphene-based mid-IR localized SPR (LSPR) biosensing platforms [96].

#### Chemiluminescence and electrochemiluminescence biosensors

Chemiluminescence (CL) sensors detect light emissions released from the chemical reaction process. After the reaction of certain chemicals, CL sensors produce light without requiring an external light source. In such systems, the recognition is usually provided by specific reactive molecules, the luminophore acts as the indicator, and the sensing signal is the emitted light. This chemical process involves the excitation of molecules to higher energy states during the reaction, and then they emit photons (light) when they return to their ground state [97]. In contrast, electrochemiluminescence (ECL) sensors integrate electrochemical and chemiluminescent principles. The light-emitting reaction is started by applying an electrical voltage that triggers redox reactions on the electrode surface. Here, the recognition relies on analyte–electrode interactions, the electrode or surface-bound luminophore acts as the indicator, and the signal is the electrochemically induced light emission. These reactions generate excited states that emit light as they relax back to lower energy levels [98]. Therefore, the main difference between CL and ECL is how the excited states are generated: The light production in CL and ECL is based on chemical energy and electrochemical reactions, respectively. Table 1 shows a summary of analytical features for CL-based assessment of Co^2+^ and VB_12_ [99–114].

**Table 1:** Analytical features of CL-based sensing platforms of CO^2+^ and VB_12_.

Sample type/Ref.	Analyte(s)	Flow technique^a^	Sample throughput (1/h)	Reagents^c^	Total flow rate (mL/min)

	CL response time (s)	linear range	LOD	RSD (%)	RR (%)

pharmaceutical preparations [99]	Co^2+^/VB_12_	FIA	–	luminol–H_2_O_2_	7

	–	1–100 mg/L Co^2+^	0.02 mg/L Co^2+^	2.9	95.3–103.1

degradation studies of CNCbl [100]	Co^2+^	MSFIA	>180	luminol–H_2_O_2_	25

	–	0.015–5.0 µg/L	0.015 µg/L	–	–

high-purity iron sample [101]	Co^2+^	FIA	<15	luminol–H_2_O_2_	2.8

	–	0.5–100 µg/L	0.5 µg/L	4.0	–

pharmaceutical preparations [102]	Co^2+^/VB_12_	r-FIA	60	luminol immobilized on an anion exchanger and H_2_O_2_ electrogenerated	14

	–	1.0 × 10^−3^–10 mg/L	3.5 × 10^−4^ mg/L VB_12_	<3.5	–

human serum, fish tissue, egg yolk, pharmaceuticals [103]	Co^2+^/VB_12_	FIA	100	luminol-O_2_	6

	1.5–5.0	2.0 × 10^−10^–1.2 × 10^−6^ g/L VB_12_	5.0 × 10^−11^ g/L VB_12_	<5.0	92.0–107.8

pharmaceutical injections [104]	VB_12_	FIA	–	luminol–H_2_O_2_	3

	10	8.68–86.9 ng/mL	0.89 ng/mL	2.5	94.8–102.6

pharmaceutical (VB12) injections [105]	Co^2+^	FIA	60	(1,10-phenanthroline)_3_ complex on the lucigenin-periodate reaction	2.45

	–	1.0 × 10^−9^–3.0 × 10^−7^ g/mL	4.4 × 10^−10^ g/mL	2.3	–

injection ampoules [106]	VB_12_	FBL (silicon photodiode detector)	72	luminol–H_2_O_2_	11.33–11.88

	–	2.4–12.0 μg/L	0.11 µg/L	<2.2	97.8–102.1

injections, tablets [107]	Co^2+^/VB_12_	FIA	–	luminol–percarbonate (H_2_O_2_ source)	10

	2	10 –5,000 µg/L Co^2+^	9.3 µg/L	2.2–4.4	95.8, 97.7

pharmaceuticals (ampoules, tablets) [108]	Co^2+^	continuous FIA with CCD photodetector	–	luminol–percarbonate (H_2_O_2_ source)	19.8

	2	4.0–300 μg/L	0.42 µg/L	2.2, 4.2	94.7–103.5

20 μg VB_12_ tablets, multivitamin tablets [110]	VB_12_	microfluidics (chip fabricated by a soft-lithographic procedure using PDMS)	–	AuNPs-enhanced luminol-AgNO_3_	0.03

	–	0.25–100 ng/mL	0.04 ng/mL	1.56	93.0–105.5 (VB_12_ tablets), 98.5–103.7 (multivitamin tablets)

egg yolk [111]	Co^2+^/VB_12_	–^b^	–	DBS, LDHs, H_2_O_2_	–

	4.0–4.5	1.0 ng/mL–5.0 μg/mL VB_12_	0.57 ng/mL VB_12_	2.8	96.0–103.0

pharmaceutical preparations [112]	VB_12_	bead injection with multicommutation	11	luminol, H_2_O_2_, Dowex 1 × 8 beads	2.6
	–	1.7–50 µg/L	0.5 µg/L	5.3	92.0–103.2

pharmaceuticals, human serum, egg yolk, fish tissue [113]	Co^2+^/VB_12_	liquid system, chitosan membrane adsorption	–	luminol–H_2_O_2_	–
	–	0.4 pg/L–40 µg/L	4.0 fg/L Co^2+^	–	–

energy drinks [114]	VB_12_	dipstick-based immunochemiluminescence	–	VB_12_ antibody, VB_12_–alkaline phosphatase conjugate, CDP-Star substrate, Tween-20, EDC-NHS, XAD-2 amberlite	–

	5	1–500 ng/mL	1.0 ng/mL	<0.2	90.0–99.3

^a^r-FIA: reversed-flow injection analysis, MSFIA: multisyringe flow injection analysis, FBL: flow–batch luminometer, CCD: charge-coupled device, PDMS: polydimethyl siloxane; ^b^the primary focus is on the novel CL amplifier (DBS–LDHs) for enhancing the detection of Co^2+^ released from VB_12_, improving selectivity and sensitivity without specifying the flow system involved in sample delivery and reaction; ^c^DBS: dodecylbenzene sulfonate, LDHs: layered double hydroxides, CDP-Star: disodium 2-chloro-5-(4-methoxyspiro{1,2-dioxetane-3,2′-5-chlorotricyclo[3.3.1.1^3,7^]decan}-4-yl)-1-phenyl phosphate, NHS: *N*-hydroxysuccinimide, EDC: ethyl-3-(3-dimethylaminopropyl)carbodiimide.

Qin et al. designed a CL sensor to detect VB_12_, incorporating flow injection analysis, where the catalytic activity of Co^2+^ released from the VB_12_ structure acted as the recognition element, luminol served as the indicator luminophore, and the sensing signal was the chemiluminescent emission triggered by H_2_O_2_. The sensor operates according to the catalytic action of Co^2+^, which is released from the VB_12_ structure through acid treatment, on the CL reaction involving luminol (which is electrostatically immobilized on an anion-exchange column) and hydrogen peroxide (H_2_O_2_). The H_2_O_2_ is electrochemically produced in real-time by utilizing a negative bias to an electrode, which converts dissolved oxygen in the flow cell. The linear range, LOD, and RSD for the detection of VB_12_ were 0.001–10 mg/L, 0.0035 mg/L, and less than 3.5%, respectively [102]. Also, Kumar et al. could well detect VB_12_ in multivitamin capsules, VB_12_ tablets, and VB_12_ injections by fabricating a sensitive CL sensor based on the reaction of VB_12_ and luminol under alkaline conditions using the carbonate enhancement effect, where VB_12_ acted as the recognition analyte, luminol as the indicator, and the CL light emission as the signal. Linear range, LOD, RSD, and RR were 5 pg/mL, 10 pg/mL to 1.0 μg/mL, 0.30–1.09%, and 97.0–99.2%, respectively. The developed approach suggested remarkable benefits, including simplicity, lower reagent use, improved sensitivity and analytical efficiency, and ease of implementation [22]. Moreover, Lok et al. assessed VB_12_ doses in a continuous-flow lab-on-a-chip system based on luminol-peroxide CL tests to monitor Co^2+^ in the molecular structure of VB_12_ [24], where the released Co^2+^ acted as the recognition target, luminol as the indicator luminophore, and the CL detected in the microchannels as the sensing output. The device includes two micromixers and a double spiral microchannel for optical detection, operating in two modes, namely, “mode I” with direct in-chip acidification and “mode II” with pre-detection external acidification. In mode I, the VB_12_ sample undergoes direct acidification within the microfluidic device. Through separate inlets (A, B, C, D, and E), the system receives, respectively, the VB_12_ sample, HCl, NaOH, luminol, and H_2_O_2_. The process begins with mixing the VB_12_ sample with diluted HCl in a designated acidification channel to release Co^2+^. Subsequently, NaOH is added to neutralize the acid mix. This neutralized sample is then integrated with luminol and H_2_O_2_ in a reaction channel, where the resulting CL signal is detected. In mode II, acidification happens outside the device. Inlets A and B are closed off, and the externally acidified VB_12_ sample and luminol and H_2_O_2_ are introduced, respectively, via inlets C, D, and E. These components mix in the reaction channel, initiating the CL detection process. Mode I achieved a linear range of 1.0 ng/mL to 10 μg/mL and an LOD of 0.368 pg/mL, requiring 30 μL samples and 3.6 s for analysis. Mode II extended the linear range to 0.10 ng/mL with an LOD of 0.576 pg mL, requiring 50 μL samples and 6 s. Mode II could effectively detect VB_12_ in nutritional supplements and egg yolks [24].

Another research group earlier introduced a CL technique for VB_12_ measurement, utilizing the Co^2+^-enhanced CL reaction between luminol and dissolved oxygen within a flow injection setup, in which Co^2+^ released from VB_12_ acted as the recognition element, luminol acted as the indicator, and the increased CL intensity represented the sensing signal. The increase in CL intensity matched the VB_12_ concentration, showing a linear response from 2.0 × 10^−10^ to 1.2 × 10^−6^ g/L and a low LOD (5.0 × 10^−11^ g/L). The results were obtained within 0.5 min at a 2.0 mL/min flow rate and displayed less than 5.0% RSD. This biosensing platform was effectively employed to analyze VB_12_ in various samples, including pharmaceuticals, human serum, egg yolk, and fish tissue [103]. Akbay and Gök also assessed CL intensities via a flow injection system for quantifying VB_12_. This technique utilized the catalytic role of Co^2+^ present in VB_12_ to facilitate the CL reaction between luminol and H_2_O_2_ under alkaline conditions, where Co^2+^ functioned as the recognition target, luminol acted as the indicator luminophore, and the CL emission served as the output signal. The enhancement in CL intensity is directly linked to the VB_12_ concentration, with an LOD of 0.89 ng/mL. CL measurements using a flow rate of 3.0 mL/min were rapid (10 s) and showed high precision (RSD < 2.5%). This method could be effectively utilized to measure VB_12_ levels in pharmaceutical injections [104]. A flow injection CL technique to detect Co^2+^ was also developed by Du et al. [105] based on the significant catalytic impact of a Co^2+^–(1,10-phenanthroline)_3_ complex on the lucigenin-periodate reaction within an alkaline environment, where the Co^2+^ complex acted as the recognition unit, lucigenin served as the indicator, and the chemiluminescent response was the sensing signal. The CL emission under optimal conditions exhibited a direct linear relationship with Co^2+^ concentrations from 1.0 × 10^−9^ to 3.0 × 10^−7^ g/mL and an LOD of 4.4 × 10^−10^ g/mL with 2.3% RSD [105]. Andrade et al. designed a flow–batch methodology paired with a large-area silicon photodiode instead of a photomultiplier tube, resulting in a convenient and automated luminometer for CL analysis. In this platform, VB_12_ served as the recognition analyte, luminol acted as the indicator, and the detected CL emission represented the signal. This system was applied to measure VB_12_ in injection ampoules, achieving detection and quantification limits of 0.11 and 0.36 μg/L, respectively. The RR, RSD, and sample measuring capacity were 97.8–102.1%, less than 2.2%, and 72 samples per hour, respectively. The authors claimed that this system featured straightforward design, adaptability, and multifunctionality, coupled with reduced usage of reagents and samples, and generated minimal waste [106].

In two distinct studies, Murillo Pulgarín et al. evaluated the chemiluminescent determination of VB_12_ [107–108]. First, a charge-coupled device (CCD) photodetector alongside UV persulfate oxidation within a streamlined continuous flow system was employed for the catalytic enhancement of Co^2+^ in the reaction between luminol and percarbonate (H_2_O_2_ source) in an alkaline setting, where Co^2+^ released from VB_12_ acted as the recognition element, luminol served as the indicator, and the CL emission intensity represented the sensing signal. UV irradiation in the persulfate environment led to the liberation of Co^2+^ from the VB_12_ structure, and the CCD detector integrated into the flow cell captured comprehensive spectral data of the Co^2+^-enhanced luminol–percarbonate reaction. A remarkable correlation between VB_12_ concentration and emission intensity was observed by achieving an LOD of 9.3 μg/L [107]. Second, the authors utilized the enhancing effect of Co^2+^ extracted with microwave assistance on the CL reaction between luminol and percarbonate, in which Co^2+^ again functioned as the recognition target, luminol acted as the indicator, and the measurable light output was the signal. Spectral analysis of the Co^2+^-catalyzed luminol–percarbonate reaction was conducted using a CCD photodetector connected to a straightforward continuous flow system. The optimal operating conditions to attain peak CL emission included 8.0 mM luminol in a 0.075 M carbonate buffer (pH 10.0), 0.15 M sodium percarbonate, a flow rate of 0.33 mL/s, and an integration time of 2 s. Not only an LOD of 0.42 µg/L for Co^2+^ concentration was obtained, but the authors also reported that this method was effectively used to quantify VB_12_ in pharmaceutical (ampoules and tablets) products formulated with OHCbl and CNCbl [108]. Kumudha and Sarada assessed the total content of VB_12_ (42 μg/100 g; Ado-Cbl as the main isomer) purified from the halotolerant green alga, *Dunaliella salina* V-101 using a luminometer and in a polystyrene cuvette, where VB_12_ served as the recognition analyte, the luminol-based reaction acted as the indicator system, and the recorded chemiluminescence was the sensing output. The results showed a high association with the value determined by the AuNPs-based RNA aptamer (40 μg/100 g) and the microbiological method (49 μg/100 g) [109].

An advanced CL system integrated with a microfluidic platform was engineered for the quantification of VB_12_, utilizing the chemiluminescent reaction between luminol and AgNO_3_, catalyzed by AuNPs, where VB_12_ served as the recognition analyte, luminol acted as the indicator luminophore, and the strong CL emission revealed the sensing signal. In this system, Ag^+^ served as a chemiluminescent oxidant, facilitating luminol oxidation in the catalytic presence of AuNPs, thereby generating a potent CL emission. The catalytic action of AuNPs enables the conversion of luminol to luminol radicals via interaction with AgNO_3_, which subsequently reacts with dissolved oxygen to produce CL emission. This innovative CL technique was successfully applied to ascertain VB_12_ doses in pharmaceutical tablets and multivitamin formulations [110]. To overcome the low selectivity of VB_12_ detection because of metal ion interference in the luminol system, Zhang et al. utilized the dodecylbenzene sulfonate-modified layered double hydroxide to catalyze a Fenton-like, ultraweak CL reaction for specifically detecting VB_12_ in egg yolk [111], where the surfactant-modified catalyst served as the recognition site, the peroxide reaction acted as the indicator system, and the ultraweak CL emission was the analytical signal. They determined the linear range (from 1.0 ng/mL to 5.0 μg/mL) and the LOD (0.57 ng/mL) for VB_12_ detection, which could be effectively used to measure its content in egg yolk. The accuracy of this novel CL method without relying on luminol was validated against the standard inductively coupled plasma-MS (ICP-MS), indicating a highly selective VB_12_ determination [111]. Domínguez-Romero et al. introduced a novel application of bead injection within multicommutation-based flow systems, featuring a surface-renewable CL flow sensor that utilizes the CL reaction between luminol and H_2_O_2_, in which Co^2+^ released from VB_12_ acted as the recognition element, luminol served as the indicator, and the Co^2+^-enhanced CL emission was the sensing signal. This technique was implemented for three commercial mineral water samples spiked with Co^2+^. The method’s effectiveness was also demonstrated by detecting VB_12_ in injectable pharmaceuticals through Co^2+^-induced CL signal enhancement in an alkaline medium, after mineralization to release Co^2+^. This biosensing platform showed a linear range of 1.7 to 50 µg/L, an LOD of 0.5 µg/L, and a sampling rate of 11 samples per hour. The data acquired through this method were rigorously validated against ICP-MS results [112]. The CL potential of luminol for the determination of Co^2+^adsorbed on a chitosan membrane was assessed based on the catalytic effect of these ions on the luminol–H_2_O_2_ CL reaction, where the chitosan membrane provided the recognition surface, luminol acted as the indicator, and the detected CL light emission was the sensing output. Linear range and LOD of Co^2+^ were found to be 0.4 pg/L to 40 μg/L and 4.0 fg/L, respectively. The membrane/liquid CL system demonstrated analytical performance comparable to the liquid-only CL system. The pre-concentration of Co^2+^ on chitosan membranes had potential for biomedical and food applications for VB_12_ determination in pharmaceuticals, human serum, egg yolk, and fish tissue [113].

**Figure 3:**
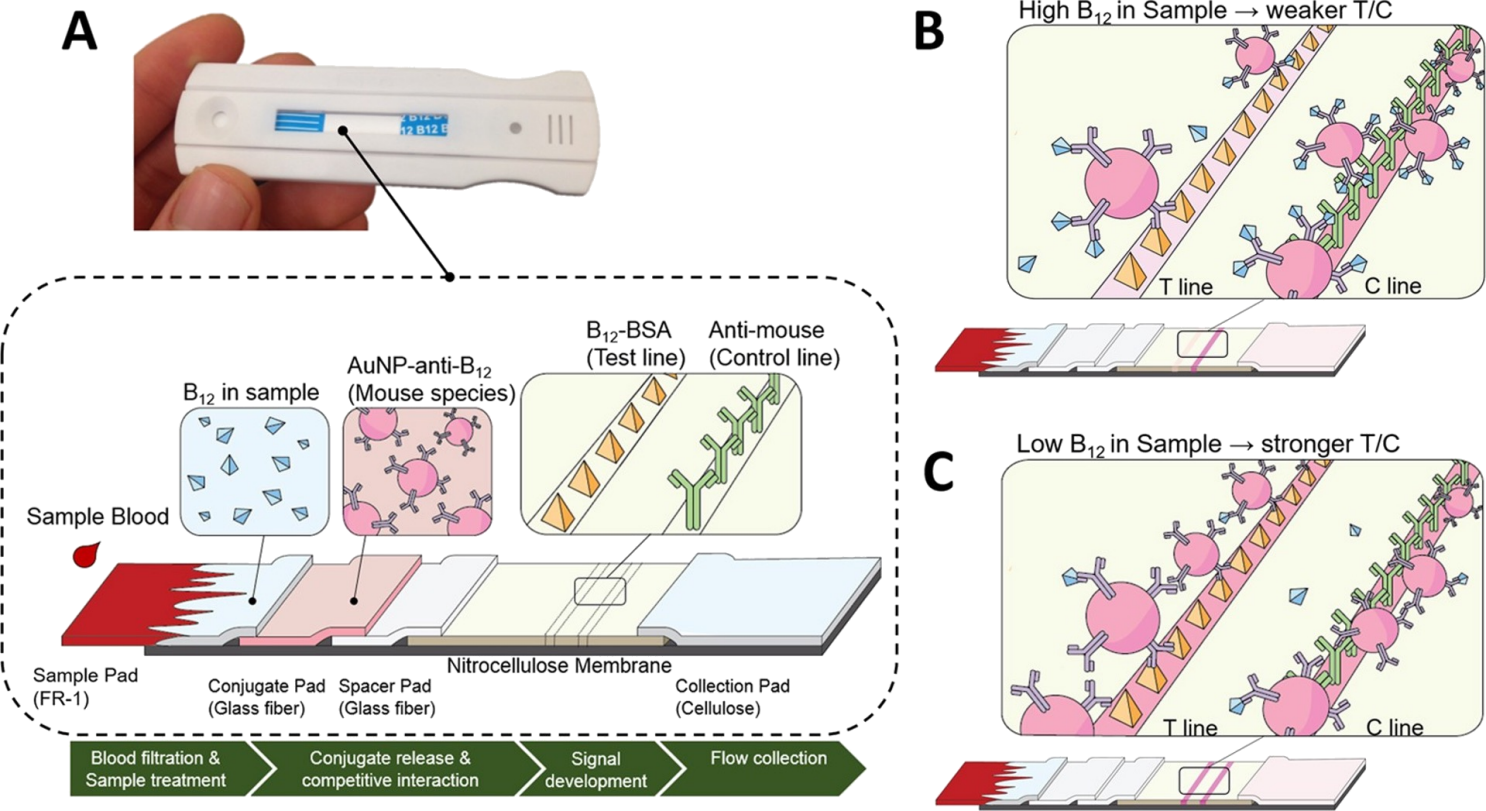
VB_12_ lateral flow test. (A) Visualization and structure of the VB_12_ test strip design and elements, (B) competitive dynamics at the test line between VB_12_ in the sample and anchored BSA-VB_12_ for restricted binding zones on AuNP-anti-VB_12_, yielding low T/C signal intensity with elevated VB_12_ levels, and (C) high T/C signal intensity with low VB_12_ doses. Figure 3 was reproduced from [115] (© 2016 S. Lee et al., published by Springer Nature, distributed under the terms of the Creative Commons Attribution 4.0 International License, https://creativecommons.org/licenses/by/4.0).

Selvakumar and Thakur utilized a dipstick-based immuno-CL biosensor to detect VB_12_ in two different energy drinks, where the antibody–antigen interaction provided the recognition event, the chemiluminescent substrate (CDP-Star) acted as the indicator, and the sensing signal was the measured photon emission. In this competitive assay, VB_12_ antibodies were fixed onto a nitrocellulose membrane, then exposed to both VB_12_ and its enzyme-bound form for competitive binding. The detection principle was based on the utilization of CDP-Star, with the generated light being inversely proportional to the VB_12_ levels. The LOD and RR of VB_12_ using this sensor were 1.0 ng/mL and 90–99.4%, respectively. Results obtained from the dipstick technique coupled with CL showed a high association with the ELISA findings [114]. Lee et al. developed NutriPhone as a quick tool to check VB_12_ levels in blood within 15 min using a smartphone, where VB_12_ acted as the recognition analyte, the AuNP–antibody conjugates and chemiluminescent reaction served as the indicator system, and the measurable optical/CL response was the sensing signal. It consisted of a special phone attachment, an app, and a test strip that could detect very low levels of VB_12_. A unique part of the test is a “spacer pad” to make the results clearer. The NutriPhone was also used in a study with twelve people, accurately determining their VB_12_ blood levels from just a tiny drop of blood from a finger prick. For this work, the serum after a 1 h incubation was separated by centrifugation (2,000 rpm, 10 min), and serum VB_12_ levels were evaluated using a chemiluminescence immunoassay. The Siemens VB_12_ kit indicated that the normal range for VB_12_ levels was between 142 and 724 pmol/L, with an LOD of 92 pmol/L [115]. The customized VB_12_ test strip detailed in Figure 3A is engineered for a lateral flow assay incorporating a blood filtration membrane, a pad for AuNP–anti-VB_12_ conjugates, a spacer pad to extend sample–conjugate interaction, a nitrocellulose membrane for VB_12_–bovine serum albumin (BSA) conjugation, secondary antibodies as test and control lines, and an absorbent pad for waste. Designed for the small molecular size of VB_12_, this structure ensures its competitive binding to antibodies. A crucial step involves the sample’s interaction with AuNP–anti-VB_12_ on the conjugate pad, significantly enhanced by the spacer pad that delays the flow to the nitrocellulose membrane, crucial for detecting low VB_12_ concentrations. With high VB_12_ levels, most conjugates bind to VB_12_ in the sample, resulting in a minimal color change at the test line (T) but a strong control line (C), leading to low T/C ratios (Figure 3B). In contrast, low VB_12_ levels yield an intense color at the test line and a weak control signal, indicating fewer conjugates reach the control line, thus high T/C ratios (Figure 3C) [115].

In a recent observational cohort study involving 11,549 pregnant women, researchers investigated the correlation between imbalances in serum folate and VB_12_, measured by chemiluminescent immunoassays, and adverse pregnancy outcomes, where folate and VB_12_ acted as the recognition analytes, the chemiluminescent immunoassay kit served as the indicator platform, and the photon emission intensity was the recorded signal. Per the manufacturer’s guidelines, the LODs of serum folate and VB_12_ were under 2 μg/L and below 50 ng/L, respectively, while the corresponding normal ranges for a healthy population were considered 5.9–24.8 μg/L and 180–914 ng/L, respectively [116]. Wiesholler et al. developed a self-assembled nanoengineered interface to facilitate direct detection of VB_12_ in serum through a simple luminescence method. Here, VB_12_ in serum was the recognition target, thulium (Tm^3+^)-doped sodium yttrium fluoride (NaYF_4_) upconversion nanoparticles (UCNPs) on gold nanotriangle arrays acted as the indicator system, and the enhanced near-infrared (NIR)-excited UV luminescence was the sensing signal. This approach relied on the synergistic effects of Tm^3+^-doped NaYF_4_ UCNPs on gold nanotriangle arrays created via nanosphere lithography on a glass slide. It greatly improved the conversion of near-NIR light to UV light, achieving a notable increase in UV light intensity (i.e., six times higher at 344 nm) when stimulated with a NIR light at 980 nm using a low power density of about 13 W/cm^2^. The process optimization was particularly advantageous for luminescence-based sensing in biological samples, as it minimized autofluorescence through NIR excitation. This methodology enabled the measurement of VB_12_ levels in serum with an LOD as low as 3.0 nmol/L from a small sample volume of 200 μL [117].

In general, there are fewer and newer studies on the ECL sensors compared to CL sensors to detect VB_12_. Bhaiyya et al. have recently fabricated a novel laser-induced graphene (LIG)-based ECL system with two and three channels to detect H_2_O_2_, VB_12_, and vitamin C (VC) from real samples. VB_12_ and VC acted as the recognition analytes, the luminophore–graphene interface served as the indicator platform, and the smartphone-captured ECL light emission was the sensing signal. This system, made from polyimide sheets capable of producing graphitized areas in one step, utilized a CO_2_ IR laser with optimized settings to construct closed bipolar electrodes (C-BPEs) and driving electrodes (DEs). A compact, 3D-printed setup was designed to support the device and integrated a smartphone, transforming it into a self-contained ECL detection platform. The smartphone captured the ECL signals and also powered the device via a DC-to-DC buck-boost converter. LIG-C-BPE-ECL-based devices with two and three channels were created to detect either single analytes or two analytes simultaneously. Linear range and LOD of H_2_O_2_, VB_12_, and VC in a two-channel LIG-C-BPE-ECL system were 0.5–100 μM and 0.303 μM, 0.5–1000 nM and 0.109 nM, and 1–1000 μM and 0.96 μM, respectively. The concurrent determination of VB_12_ and VC was possible using a three-channel version of this portable and versatile system [118]. These researchers, in another study, reported ECL sensing of VB_12_ using LIG-based BPE and single electrodes (SEs) in a 3D-printed portable system, where VB_12_ acted as the recognition target, the graphene electrode served as the indicator, and the electrochemically triggered luminescence resulted in the output signal. Similar to the previous investigation, they utilized an Android smartphone for data acquisition, including operation of the DC-to-DC buck-boost converter and the capture of ECL images. Linear range and LOD of VB_12_ in BPE and SE modes were 0.5–700 nM and 107 pM, and 0.5–1000 nM and 94 pM, respectively [119].

### Fluorescence-based biosensors

#### Small-molecule-based fluorescent chemosensors

Fluorescence-based chemosensors are highly sensitive, specific, and rapid tools to detect B-group vitamins [84,120]. Xu et al. prepared an innovative strategy by integrating Förster resonance energy transfer (FRET) and flow injection analysis to assess VB_12_ based on its fluorescence quenching (FQ) on the acridine orange/rhodamine 6G system. In this assay, VB_12_, the dye pair acridine orange/rhodamine 6G, and fluorescence quenching acted as the recognition analyte, the indicator, and the sensing signal, respectively. Energy transfer between acridine orange and rhodamine 6G within dodecyl benzene sodium sulfonate was effectively facilitated using a 454 nm argon laser as the light source, significantly enhancing rhodamine 6G’s fluorescence emission. However, the presence of VB_12_ was observed to drastically reduce fluorescence in this setup. Utilizing a combined solution of acridine orange, rhodamine 6G, and dodecyl benzene sodium, and introducing VB_12_ into this mixture, resulted in distinctive negative peaks useful for VB_12_ determination. Linear range and LOD of VB_12_ were assessed to be 0.04–2.0 µM and 1.65 µM, respectively. This fluorescent chemosensor could be successfully employed to detect VB_12_ in injections [121]. A new fluorescent probe composed of 7‐nitrobenzo‐2‐oxa‐1, 3‐diazole (HINBD) skeleton was also developed to measure VB_12_ in medical drugs. Here, VB_12_ was the target molecule, fluorescence of HINBD represented the indicator, while its suppression provided the output signal. This soluble probe in water showed a robust emission within the visible spectrum (excitation wavelength λ_ex_ = 479 nm and emission wavelength λ_em_ = 545 nm) and maintained stability across various pH levels. The presence of VB_12_ was observed to significantly suppress HINBD’s fluorescence, with the degree of suppression directly correlating to the VB_12_ dose. Under ideal conditions, a linear detection span from 0 to 24 nmol/L was established, with an LOD of 83 pmol/L. Scholars also reported reliable outcomes in analyzing the concentration of this water-soluble vitamin in pharmaceutical products [122]. In a distinct work, Chakravarty et al. designed a real-time, label-free opto-electrical (fluorescence and impedance) sensing system of VB_12_ using silk fibroin protein from *Bombyx mori*. The recognition was based on VB_12_ binding, micropatterned silk fibroin films functioned as the indicator, and fluorescence/impedance variations were the readout signals. The solution and films’ inherent autofluorescence led to an LOD of 3.0 pg/µL, while the impedance-based detection resulted in an LOD of 17.8 ppm and 0.25 ppm of VB_12_ in aqueous solutions and human blood serum, respectively [123]. Wang et al. introduced a new approach for synthesizing fluorescent nanowires from nanoscale diamonds. In their FRET-based sensor, VB_12_ served as the analyte, sulfur-modified diamond nanowires acted as the indicator, and the fluorescence response was recorded as the signal. These nanomaterials are recognized for their exceptional chemical capabilities alongside their dependence on size, shape, and constituent materials. Using a thermal process that combined oxidized nanoscale diamonds with dibenzyl disulfide at 900 °C, researchers successfully fabricated sulfur-modified nanoscale diamonds. A method involving porous anodic aluminum oxide templates and cathodic electrophoretic deposition was utilized to fabricate the sulfur-modified nanoscale diamond nanowires. Eventually, an optical sensor based on the sulfur-modified nanoscale diamond nanowires was developed, demonstrating exceptional sensitivity and specificity in detecting VB_12_ through the FRET mechanism [124]. In an intriguing work, Ahmad et al. applied FRET-based genetically encoded nanosensors, dubbed SenVitAL (“Sensor for Vitamin Anemia Linked”), as a refined approach for real-time monitoring of metabolite levels of VB_12_ directly within live cells using a construct that combines the VB_12_ binding protein (BtuF) with cyan (CFP) and yellow (YFP) fluorescent protein variants. In this design, BtuF served as the recognition element, CFP/YFP functioned as the indicator pair, and changes in FRET efficiency represented the measurable signal. Transferred into various expression vectors, SenVitAL demonstrated high specificity for VB_12_, maintained stability across pH variations, and quantified its concentrations in a dose-dependent manner, with an apparent affinity of ≈157 μM [125].

#### Nanocluster/nanoparticle-based fluorescent sensors

The utilization of nanocluster-based sensors in measuring VB_12_ is pivotal for improving sensitivity and specificity in food and pharmaceutical analysis. These sensors exploit the unique optical and electronic properties of these nanostructures to achieve low LODs and high accuracy, which are necessary in ensuring the nutritional adequacy and safety of biofunctional products [126–130]. A highly sensitive approach was successfully designed based on the FQ of BSA-stabilized gold nanoclusters (AuNCs) for the rapid determination of VB_12_, where VB12, AuNCs, and the quenched fluorescence were the recognition analyte, indicator, and signal, respectively. The system worked within the concentration range of 0.16–38.5 μg/mL with an LOD of 0.1 μg/mL. A 97.7–102% RR with an RSD of 2.0–5.9% was obtained when this sensor was utilized to analyze VB_12_ in commercially available injectable dosage products [126]. A new fluorescent sensing probe without using toxic organic solvents was developed based on a histidine-stabilized copper nanocluster for the detection of VB_12_. A remarkable reduction in the emission intensity of the fabricated probe with the addition of VB_12_ was found. This quenching occurred because of FRET between the analyte and probe. This method could selectively assess VB_12_ with an LOD of 3.30 × 10^−9^ mol/L amid other potentially interfering vitamins [127]. Another study was performed to find a response to how metal ions interact with AuNCs, causing structural alterations in the clusters that manifest as changes in fluorescence, enabling the detection of heavy metals like cobalt and cadmium. Here, the bound metal ions acted as the recognition elements, BSA-derived AuNCs provided the indicator, and the fluorescence modulation via intersystem crossing and FRET was the measurable signal. This mechanism facilitated the sensitive detection of cobalt and cadmium within a concentration range of 5–165 ng/mL and 20–1000 ng/mL, respectively. Furthermore, this technique was effectively utilized to quantify VB_12_ in commercial injections [128]. Silver nanoclusters emitting red light within lysozyme structures were fabricated through the reduction of dithiothreitol and utilized to detect copper ions and VB_12_. The quenching mechanism induced by VB_12_ was based on the inner filter effect (IFE) and FRET. These homogenous nanoclusters with excellent water solubility and photoluminescence (PL) potential could be applied to detect VB_12_ in real samples [129]. Qu et al. also reported that VB_12_, via the IFE mechanism, quenched fluorescence induced by silver nanoclusters (AgNCs) capped by hyperbranched polyethyleneimine (HBPEI) with various molecular weights and terminal groups. Analyte, indicator, and signal were VB_12_, HBPEI-stabilized AgNCs, and fluorescence quenching, respectively. Silver nanoclusters templated by a 600 kDa HBPEI as a sensitive probe could be used to monitor VB_12_ in a linear range of 0.005–70 μM and an LOD of 0.00262 μM. The sensing potential was also validated by detecting this vitamin with desired RRs in tablets (97.13–102.58%) and injections (99.09–105.09%) [130]. Recently, Zhang et al. evaluated the capability of stable histidine-modified silver nanoclusters as a blue fluorescence emission probe for measuring VB_12_. Histidine and ascorbic acid were utilized as capping and reducing agents, respectively. Linear range and LOD of VB_12_, respectively, were 0.5–200 μM and 0.038 μM under optimized conditions of histidine (5 mL) and ascorbic acid (200 μL) volumes, pH (5.0), temperature (55 °C), and incubation time (5 h). The successful application of this nanoprobe in real-sample analysis and temperature measurement confirmed its performance for effective VB_12_ assessment, highlighting an innovative strategy with excellent sensitivity, selectivity, and practical utility [131].

Yu et al. created a fluorescent probe based on water-soluble copper nanoclusters shielded by PEI through the synergistic process of UV radiation and microwave heating. The sensor’s FQ mechanism relied on the integration of FRET and IFE, where VB_12_ acted as the recognition analyte, the PEI–Cu nanoclusters served as the indicator, and the quenching of fluorescence provided the measurable signal. It was applied to monitor tetracycline hydrochloride and VB_12_ within linear concentration ranges of 0.33–66.67 μmol/L and 0.33–53.33 μmol/L, respectively. The LOD and limit of quantification were estimated to be 55.50 and 56.34 nmol/L as well as 184.82 and 187.61 nmol/L for tetracycline hydrochloride and VB_12_, respectively. This probe, compared to HPLC analysis, presented satisfactory results for measuring VB_12_ in oral liquid or tablets [132]. Through a single-step wet chemical synthesis process, Hu et al. also produced an AuNC-based fluorescent sensor by combining silver addition and dual ligands (thiosalicylic acid (TSA) and BSA). These ligands were mixed drop by drop into a vial at 80 °C for 2 h. They studied the effect of various aromatic thiols and the molar ratio of gold (Au) to silver (Ag) on the nanoclusters’ PL efficiency. These alloy nanoclusters proved their potential as effective fluorescent sensors for assaying VB_12_ and chlortetracycline hydrochloride (CCH), as the fluorescence induced by these nanoclusters could be quenched upon adding the investigated analytes due to the combined effects of FRET and IFE. The LOD for VB_12_ and CCH were, respectively, determined to be 0.071 μmol/L and 0.064 nmol/L within the linear range of 0.33–60 μmol/L. They pointed out that this fluorescent sensor was capable of effectively detecting VB_12_ in mineral water and tablets, as well as CCH in veterinary medications and topical creams [133]. It was mentioned that histidine-modified silver nanoclusters [131], copper nanoclusters protected by PEI [132], and TSA/BSA-Au/AgNCs [133] can also be considered highly efficient temperature sensors.

Chau et al. have recently designed a fluorescent assessment for VB_12_ detection through the IFE of 1,3-propanedithiol-functionalized silver nanoparticles (PDT-AgNPs). Here, VB_12_ served as the recognition target, PDT-AgNPs acted as the fluorescent indicator, and the IFE quenching was the sensing output. The authors initially synthesized AgNPs via the reduction of Ag^+^ to Ag^0^ by applying NaBH_4_. The fluorescence intensity (λ_ex_ = 360 nm and λ_em_ = 410 nm) could be substantially enhanced by functionalizing AgNPs with PDT for 2 h, resulting in a significant FQ in the presence of VB_12_ due to spectral overlap. Here, VB_12_ served as the recognition target, PDT-AgNPs acted as the fluorescent indicator, and the IFE quenching was the sensing output. Linear range and LOD of VB_12_ were determined to be 1–50 μM and 0.5 μM, respectively. The favorable sensitivity and selectivity of the developed PDT-AgNPs-based fluorescent probes were also affirmed by accurately quantifying VB_12_ in pharmaceutical tablets [134]. In another study, researchers made a composite from silicon NPs (SiNPs) and AuNPs as a selective fluorescent probe (λ_ex_ = 420 nm and λ_em_ = 520 nm) to sensitively detect Co^2+^ and VB_12_ by integrating selective aggregation and IFE. Green-emitting SiNPs were prepared using a one-pot hydrothermal method and then functionalized with thioglycolic acid and cetyltrimethylammonium bromide. Selective aggregation of AuNPs occurred in the presence of Co^2+^ and VB_12_, enhancing the LSPR absorption at 520 nm and significantly quenching the green fluorescence of SiNPs through IFE. A linear FQ efficiency with Co^2+^ doses was recorded in the range of 0.1 to 80 µM, achieving an LOD of 60 nM, which was lower than the guideline value of Co^2+^ in drinking water (1.7 µM). Also, linear range and LOD for VB_12_ were assessed to be 0.1–100 µM and 69 nM, respectively [135].

Gholami et al. also synthesized a graphene oxide (GO) nanolayer for the label-free detection of VB_12_ using fluorescence spectroscopy. The developed nanolayer showed high selectivity as it could specifically interact with VB_12_ to discriminate it from some vitamins (such as vitamins B_1_, B_6_, and B_9_, and VC), as well as other substances (i.e., lauric acid, glucose, urea, and uric acid). The detection mechanism was based on the quenching effect of VB_12_ on the nanolayer’s fluorescence emission. In this case, the selective interaction of VB_12_ with GO represented the recognition event, the intrinsic fluorescence of the GO nanolayer was the indicator, and the quenching effect constituted the signal. Linear range and LOD of VB_12_ were 2.5 × 10^−7^–2.81 × 10^−5^ M and 3.2 × 10^−7^ M, respectively [136].

#### Carbon dot-based fluorescent biosensors for vitamin B_12_

Fluorimetric assays using organic dyes [137–139], semiconductor quantum dots (scQDs) [138,140], and metallic nanoclusters [131,141] were earlier suggested to analyze VB_12_. However, there are some significant concerns in utilizing organic dyes (e.g., photobleaching, limited photostability, and environmental sensitivity) [142–144], scQDs (e.g., toxicity, use in size-sensitive applications, low solubility, and batch-to-batch variations in biological environments) [145–148], and metallic nanoclusters (e.g., high reactivity and cytotoxicity in biological applications, difficulties in precise size control, low quantum yield, and low-purity synthesis) [149–150] in biosensing of VB_12_. Carbon dots (CDs), as practical substitutes for these fluorescent components, are characterized by sizes ranging from less than 10 nm to around 60 nm [151]. While CDs typically display dot-like structures, scientists have successfully engineered these fluorescent NPs in various sizes and shapes, including triangles, ribbons, and rods, by carefully selecting precursors and designing the reaction process [152–153]. There are four different classes of CDs, including carbon quantum dots (CQDs), carbon nanodots (CNDs), graphene quantum dots (GQDs), and carbonized polymer dots (CPDs). CQDs are typically spherical and characterized by distinct crystal lattices and chemical groups on their surface, contributing to their unique optical and chemical properties. CNDs exhibit a high degree of carbonization along with the presence of some chemical groups on their surface. Unlike other carbon-based nanomaterials, they typically do not display an obvious crystal lattice structure and are characterized by the absence of polymer features. GQDs are tiny fragments of graphene, composed of a single layer or a few layers of graphene sheets, featuring distinct graphene lattice structures and chemical groups positioned along the edges or within interlayer defects. Last, CPDs feature a hybrid structure of polymer and carbon, consisting of abundant functional groups or polymer chains on the surface and a carbonized core [154–156]. CDs possess remarkable characteristics to boast versatile applications, extending from biomedical fields (e.g., bioimaging, drug delivery, and gene delivery) to various other domains (e.g., photocatalysis, photovoltaic cells, CL, ECL, optical sensors, fluorescent inks, and light-emitting diodes) [157–158]. The wide applicability of CDs is due to the superior electron conductivity, resilience against photobleaching and photoblinking, high photoluminescent quantum yield, tunable fluorescence, resistance to photo-decomposition, adjustable excitation and emission, enhanced electrocatalytic activity, high aqueous solubility, excellent biocompatibility, enduring chemical stability, low cost, negligible toxicity, and a significant surface area-to-volume ratio [152,159–160].

In recent years, more attention has been directed towards CQDs due to their advantageous features, including excellent PL properties, straightforward and economical synthesis routes, affordability of starting materials, high water solubility and chemical stability, minimal toxicity, and ease of functionalization [158,161]. CQD-based sensors and biosensors operate through a variety of mechanisms including FQ (both static and dynamic), energy transfer, IFE, photoinduced electron transfer (PET), and FRET, enabling their application in detecting a wide range of analytes such as metal ions, acids, proteins and polypeptides, DNA and miRNA, water pollutants, hematin, drugs, and vitamins [158]. There is an intense tendency to utilize CDs, especially CQDs, in biosensing and cell bioimaging studies of water-soluble vitamins such as VC [162–166] and B-group vitamins [166–170]. Table 2 summarizes a list of QD-based fluorescent sensors, their synthesis methods, sensing mechanisms, as well as optical and physical properties for detecting VB_12_ in different food and pharmaceutical samples.

**Table 2:** A summary of QDs-based fluorescent biosensors for the detection of VB_12_ in food and pharmaceutical products.

VB_12_-containing sample type/Ref.	Precursors^a^	Synthesis method	QD type^b^	Quantum yield (%)	Average size (nm)

	λ_ex_ (nm)	λ_em_ (nm)	sensing mechanism^c^	linear range (μM)	LOD (μM)

commercial injections [206]	DAN, GO	citric acid pyrolysis	GQDs	–	35–40

	328	423	IFE-based FQ	–	6.37 × 10^‒6^

Tap/(Commercial) drinking water (Co^2+^ ions) [208]	citric acid, cysteamine·HCl	hydrothermal (160 °C, 4 h)	N,S-GQDs	–	3

	345	425	FQ by Co^2+^–ligand complexation	0–40	1.25

injection, saliva, fetal bovine serum [210]	TCBQ, EDA	Schiff base condensation	CPDs	2.96	0.6–2.1

	350	440	IFE-based FQ	25–100	0.14

tablets, human urine [213]	glycerol, trisodium citrate, APTES	microwave process (400 W, 180 °C, 10 min)	SiQDs	10.5	4–5

	360	460	IFE-based FQ	0.5–16	0.158

drug tablets [214]	sodium oxalate, citric acid, DAMO (Si source), catechol, thiourea	microwave-assisted hydrothermal	SiQDs	25	6–8

	420	520	IFE-based FQ	0.05–30	0.05

Water (Co^2+^ ions) [221]	PVP, AEAPDMMS	hydrothermal (100 °C, 3 h)	SiQDs	2.36	4.1

	370	435	SQE-based FQ	1–120	0.37

milk powder [222]	curryberries juice	ultrasonication	nCQDs	10.24	2–8

	360	510	PL quenching	1–40	0.04

Milk [226]	milkcap mushroom (*Lactarius hatsudake*)	hydrothermal (200 °C, 12 h)	nCQDs	22.88	3

	324	408	IFE-based FQ	0–20	36.9

drug tablets [182]	*O*-phenylenediamine, 4-aminobenzoic acid	hydrothermal (190 °C, 3 h)	nCQDs	32	2.22

	390	567	IFE-based FQ	0–90, 140–250	0.119

drug tablets [229]	*Saccharomyces*, ethanediamine (N source)	microwave-assisted hydrothermal (200 °C, 3 h)	N,nCQDs	16	2.9

	380	460	FRET-based FQ	0–100	2.19

water (Co^2+^ ions) [230]	Kelp (C source) EDA (N dopant)	microwave irradiation (200 °C, 10 min, 800 W)	N,nCQDs	23.5	≈3.7

	370	450	IFE-based FQ	1–200	0.39

water (Co^2+^ ions) [231]	biomass quinoa saponin powder (C source), EDA (N dopant)	hydrothermal (220 °C, 10 h)	N,nCQDs	22.2	≈2.25

	390	470	FQ by Co^2+^ interaction	2–150	0.49

water (Co^2+^ ions), serum and milk (VB_12_) [232]	*Weissella* sp. Kl-3 (Gram-positive bacteria), ampicillin sodium	hydrothermal (200 °C, 6 h)	N,nCQDs	8.96	5.2

	346	424	IFE-based FQ	0–25 (VB_12_), 0–50 (Cr^6+^)	0.0515 (VB_12_), 0.10657 (Cr^6+^)

human serum [233]	bird's nest, distilled water	hydrothermal (180 °C, 5 h)	nCQDs	–	–

	386	471	dynamic electron transfer	0–100	0.24

drug tablets [176]	CDPC, EDA	hydrothermal (160 °C, 10 h)	BCQDs	22.3	2.84

	350	450	Co^2+^ ion-induced CQD aggregation and electron transfer	0.5–3.0	<0.081

VB_12_ injection (0.5 mg/mL) [181]	ʟ-tartaric acid, urea	solvothermal (180 °C, 4 h)	N-doped yellow-emitting CDs	16.7	≈8.2

	450	550	IFE-based FQ	0–200	2.045

VB_12_ powders (CNCbl, Sigma), VB_12_ injections and tablets [177]	citric acid monohydrate, nicotinamide	microwave-assisted hydrothermal (80 W, 160 °C, 20 min)	NA-CQDs	–	<10

	366	444	IFE-based FQ	0.1–60	0.0317

VB_12_ tablets, vitamin drink, human serum [178]	citric acid	hydrothermal (170 °C, 6 h)	CQDs	15.2 (liquid), 39.9 (solid)	34.03

	595	560	IFE-based FQ	0.5–100	0.06078

VB_12_ powders [179]	citric acid, sodium hydrate, quinine sulfate	thermal (300 °C, 2 h), sonication (30 min, 60 W, 40 kHz)	t-QDs	16.28	6

	330	420	FRET-based FQ	1–12^d^	<0.1^d^

VB_12_ powders [180]	hydroquinone, EDA	self-exothermic reaction driving the formation of the nanocrystalline core (dissolution- oxidation- carbonization)	CQDs	24.6	≈4

	370	525	IFE-based FQ	0.75–100	0.2

VB_12_ tablets, other cobalt-containing medicines [183]	citric acid, DETA	hydrothermal (200 °C, 5 h)	NCQDs	58	2.3

	391	438	PET-based FQ	0–90	0.4

lake water, fetal bovine serum, milk [171]	methyl-*p*-benzoquinone, triethylenetetramine	Schiff base crosslinking reaction	orange-emitting CDs	6.56	5

	460	580	IFE-based FQ	0.05–200	0.01

VB_12_ powders [173]	Safranine T, ethanol	salvothermal (200 °C, 6 h)	orange-emitting CDs	7.6	2.04

	545	595	IFE-based FQ	1–65, 70–140	0.62

pharmaceutical injections [234]	*Cannabis sativa* paste, EDA, glutathione	hydrothermal (180 °C, 16 h)	N,nCQDs	14	4–6

	320	384	SQE-based FQ	20–100^d^	7.87^d^

VB_12_ tablets and injections [174]	ammonium citrate	hydrothermal (160 °C, 6 h)	CDs	–	3.03

	350	446	IFE-based FQ	0.3–15	0.093

VB_12_ tablets, natural water (Co^2+^ ions) [175]	ʟ-cysteine	hydrothermal (300 °C, 2 h)	CDs	13.2	3.6

	325	395	PL quenching	0.01–100	0.01

tap water (Co^2+^ ions) [184]	PEI	hydrothermal (-)	CQDs	8.68	2.82

	340	462	IFE-based FQ	0.05–11	0.048

VB_12_ tablets (Co^2+^ ions) [185]	glycine (N and C source), PEI	hydrothermal (4 h at 200 °C)	NCQDs	57	2–2.5

	360	464	IFE, SQE, aggregation, complex formation between N-CQDs (amino group) and Co^2+^	0.5–3.0	0.12

VB_12_ powders (CNCbl, Sigma) [186]	TSA, DETA	hydrothermal (160 °C, 6 h)	NCQDs	43	2.7

	390	450	FRET-based FQ	0.001–20	0.00021

VB_12_ tablets, human serum, energy drink [187]	ʟ-aspartic acid, 3,6-diaminoacridine hydrochloride	hydrothermal (170 °C, 5 h)	NCQDs	22.7	3.8

	360	450	IFE-based FQ	0–70	0.05628

veterinary VB_12_ injection (0.5 mg/mL) [188]	ʟ-tartaric acid, urea, DMF	hydrothermal (180 °C, 4 h)	NCQDs	15.9	3.8

	450	550	IFE-based FQ	0–200	2.101

milk, two kinds of vitamin drinks [190]	thiamine nitrate (N and S source)	hydrothermal (180 °C, 10 h)	N,S-CQDs	10.4	1.5

	338	416	FRET-based FQ	0.33–28.30, 28.30–74.85	0.0156

VB_12_ tablets and injections [191]	*O*-phenylenediamine, thiourea	hydrothermal (180 °C, 12 h)	N,S-CQDs	14.3	≈2.9

	420	565	IFE-based FQ	0.25–20	0.775

tap water, lake water (Co^2+^ ions) [192]	ʟ-cysteine	hydrothermal (180°C, 12 h)	N,S-CQDs	27	≈6

	340	420	SQE + electron transfer	1–50	0.026

VB_12_ injections [195]	cystamine, pomegranate juice	hydrothermal (180 °C, 8 h)	N,S-CQDs	–	2–3

	340	414	–	0–110	0.082

tap water (Co^2+^ ions) [198]	freeze-dried tofu, EDA, phosphoric acid	hydrothermal (210 °C, 4 h)	N,P-CQDs	64	2.9

	360	460	SQE + IFE-based FQ	0–0.5	0.058

VB_12_ tablets, blood serums [200]	ʟ-arginine, phosphoric acid	hydrothermal (240 °C, 12 h)	N,P-CQDs	18.38	2.4

	340	444	IFE-based FQ	1.99–98.6, 98.6–176	0.059

VB_12_ tablets, vitamin drink, mineral water [8]	phenylboronic acid	hydrothermal (200 °C, 10 h)	BCDs	12	3.3

	247	323	IFE + FRET-based FQ	0.2–30	0.008

VB_12_ tablets and injections (Co^2+^ ions and VB_12_) [199]	phosphoric acid (85%), sucrose, 1,2-EDA	hydrothermal (-)	N,P-CQDs	6.88	3.44

	365	451	SQE + IFE-based FQ	2–31 (VB_12_), 1.7–12 and 28–141 (Co^2+^)	0.003 (VB_12_), 0.0294 (Co^2+^)

pharmaceutical injections (1,000 and 100 µg/mL of VB_12_) [202]	tellurium powder, sodium borohydride (NaBH_4_), cadmium chloride hydrate (CdCl_2_·2.5H_2_O), TGA	hydrothermal (90 °C, 3 h)	CdS QDs	0.23^e^	3.6

	390	523	IFE-based FQ	0.02–0.4, 1.5–70.0	0.002

blood serum, urine, pharmaceuticals (multivitamin injections) [201]	cadmium chloride hydrate (CdCl_2_·H_2_O), sodium sulfide (Na_2_S), MPA	mix, adjust pH, deaerate, add reactants, stir overnight, dilute, store	CdS QDs	–	1.72

	370	532	photoinduced charge transfer by nonradiative FRET	0.5–100^d^	6.91^d^

tap water (Co^2+^ ions) [203]	SQDs, Co^2+^, norfloxacin	mixing and reacting for 10 min at room temperature	SQDs	–	5.36

	360	≈410	ACQ-based FQ	0–90	0.02

VB_12_ powders (CNCbl, Sigma) [204]	zinc acetate dihydrate, sodium sulphide flakes, manganese acetate tetrahydrate	thermal (refluxing; 100 °C, 3 h) and mechanical (sonication)	Mn^2+^-doped ZnS QDs	0.07	4

	320	587	FRET-based FQ	4.9 × 10^‒6^– 29.4 × 10^‒6^	1.15 × 10^‒6^

several commercial health beverages, Milk, VB_12_ tablets [235]	aegle marmelos fruit juice (for CQDs), lemon juice (for surface functionalization)	ultrasound-assisted hydrothermal	N-, K-, and Ca-doped nCQDs	4.10	≈4.4

	250	290–750 (pH-dependent)	adsorption + FRET between VB_12_ and CQDs	0.01–100	9.16 × 10^−4^

^a^DAN: 1,8-diaminonaphthalene, GO: graphene oxide, APTES: (3-aminopropyl)triethoxysilane, DAMO: *N*-[3-(trimethoxysilyl)propyl]ethylenediamine, PVP: poly(vinylpyrrolidone), EDA: ethylenediamine, AEAPDMMS: 3(2-aminoethylamino)propyldimethoxymethylsilane, CDPC: cytidine diphosphate choline, TCBQ: tetrachlorobenzoquinone, DETA: diethylenetriamine, PEI: polyethyleneimine, TSA: thiosalicylic acid, DMF: dimethylformamide, TGA: thioglycolic acid, MPA: mercaptopropionic acid; ^b^GQDs: graphene quantum dots, N,S-GQDs: nitrogen and sulfur co-doped GQDs, SiQDs: silicon quantum dots, BCDs: boron quantum dots, CPDs: carbonized polymer dots, nCQDs: natural carbon-based quantum dots, NCQDs: nitrogen-doped NCQD, N,nCQDs: nitrogen-doped nCQDs, Mn^2+^-doped ZnS: manganese-doped zinc sulfide, BCQDs: biomimetic carbon quantum dots, NA-CQDs: nicotinamide-functionalized CQDs, t-QDs: thermally reduced quantum dots, N,S-CQDs: nitrogen and sulfur co-doped CQDs, N,P-CQDs: nitrogen and phosphorus co-doped CQDs, TGA-CdS QDs: cadmium telluride quantum dots, SQDs: sulfur quantum dots; ^c^FQ: fluorescence quenching, IFE: inner filter effect, SQE: static quenching effect, PL: photoluminescence quenching; PET: photoinduced electron transfer, ACQ: aggregation-caused quenching; ^d^in μg/mL; ^e^0.28 relative to fluorescein (as reference dye) with a quantum yield of 0.93 at 490 nm and in sodium borate buffer (pH 9.5).

#### Carbon dot-based fluorescent sensors

CDs that emit light at longer wavelengths have recently attracted growing interest regarding their potential applications in biology. Huang et al. studied the Schiff base crosslinking reaction between methyl-*p*-benzoquinone and triethylenetetramine to prepare orange-emitting CDs with a quantum yield of 6.56% at ambient temperature. These nanoscale materials (5 nm) revealed a robust excitation-dependent emission [171]. Thanks to the strong IFE of the as-synthesized CDs, they could effectively be applied for the sensitive determination of VB_12_ concentrations in the linear range of 50–200 μM with an LOD of 0.01 μM. Here, VB_12_ acted as the recognition analyte, the CDs were the fluorescent indicator, and IFE fluorescence quenching served as the signal. The designed fluorescent sensor by introducing various VB_12_ doses (1, 10, and 50 μM) into CDs was successfully applied to detect VB_12_ in lake water, fetal bovine serum, and milk samples. The ranges of RR and RSD values for the corresponding samples were 98.0–108.8% and 1.54–2.76%, 98.9–102.0% and 1.94–3.56%, as well as 97.9–108.0% and 1.87–3.41%, respectively. Accordingly, these nanoprobes would be promising sensing tools for measuring VB_12_ in complex sample matrices [171]. The CDs exhibited two pronounced absorption peaks near 278 and 445 nm. These peaks correspond to π–π* transitions among aromatic sp^2^ carbon atoms and the n–π* transitions among C=O/C=N bonds, respectively [171–172]. The emission characteristics of these CDs are generally linked to the quantum confinement effect or the states of their surfaces. The CDs’ modest crystallinity suggested that their fluorescence primarily originated from surface states. The CDs display an excitation-dependent fluorescence, with λ_em_ exhibiting a redshift when λ_ex_ was increased from 320 to 520 nm. At an excitation of 460 nm, the peak λ_em_ for the CDs was identified at 580 nm. Furthermore, when compared to rhodamine 6G (as a standard), the relative quantum yield of the CDs is approximately 6.56%. This results in a vibrant orange fluorescence when observed under UV light [171]. Meng et al. also developed orange-emitting CDs for the label-free identification of VB_12_. These excitation-independent CDs were synthesized from safranine T and ethanol through a simplified hydrothermal procedure. The ability of VB_12_ could well quench the fluorescence produced by CDs via the IFE mechanism, within two distinct linear detection ranges of 1–65 μM and 70–140 μM, with an LOD of 0.62 μM [173]. In another study, a new CD-based fluorescent sensor from ammonium citrate as precursor in a hydrothermal process was developed for sensing VB_12_ (LOD of 93 nM). The IFE was the main mechanism due to the overlap between the UV–vis absorption spectrum of VB_12_ and the emission/excitation spectra of the CDs. Monitoring VB_12_ in injection and tablet samples using these sensors resulted in RR and RSD values of 93.3–109.2% and 0.67–1.62%, respectively [174]. Li et al. synthesized QDs (3.6 nm) from ʟ-cysteine via a hydrothermal method at 300 °C for 2 h in order to detect Co^2+^ across a range from 0.01 to 100 μM via PL quenching [175].

#### Carbon quantum dot-based fluorescent sensors

**Figure 4:**
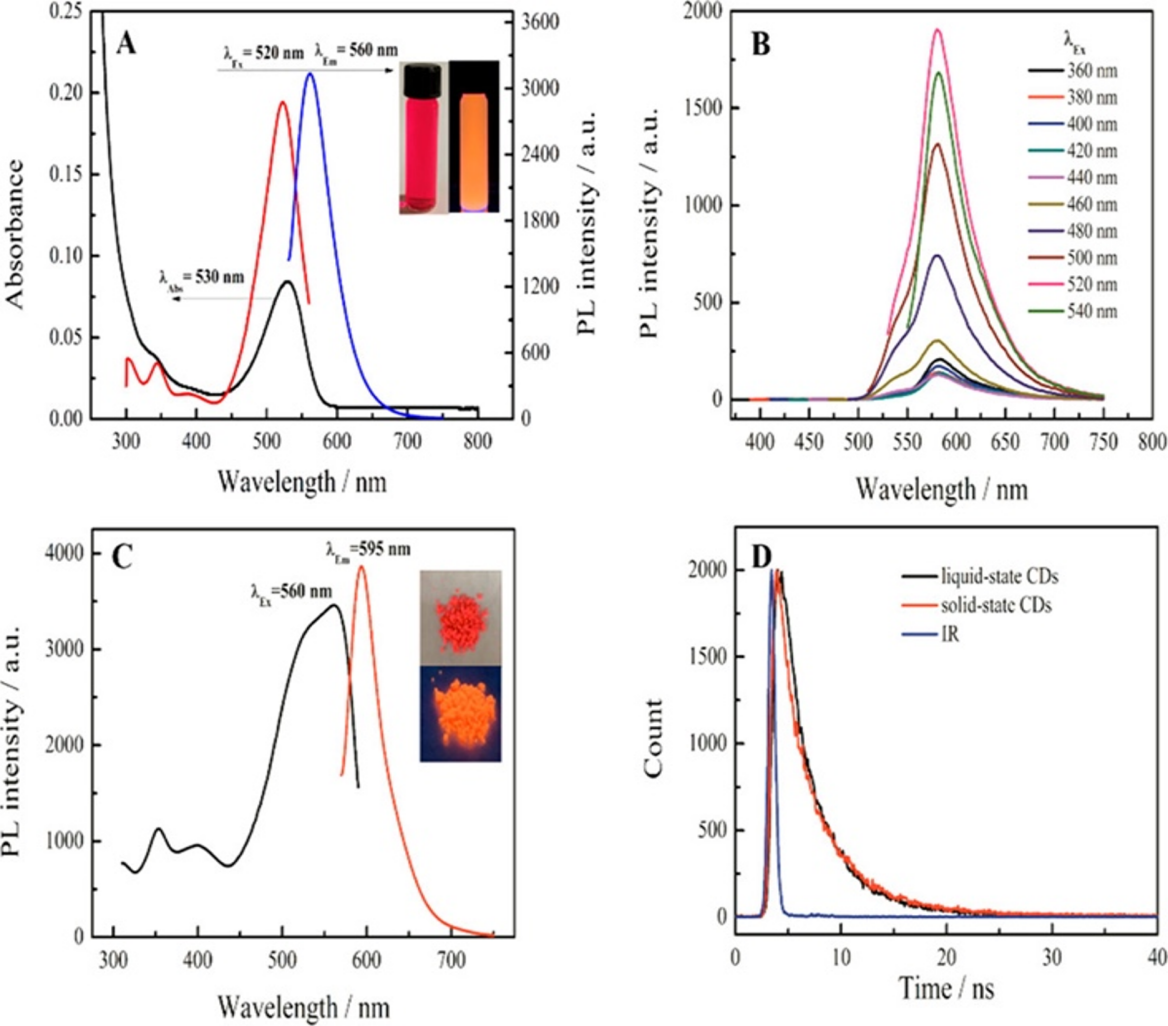
(A) Spectral characteristics including UV–vis absorption, fluorescence excitation, and emission of CDs in water (inset: daylight and 365 nm UV light exposure visuals of CDs). (B) Emission profiles of water-based CDs across excitation wavelengths from 360 to 540 nm. (C) Excitation and emission spectral data for CDs in solid form (inset: visual representation of solid-state CDs under natural and 365 nm UV illumination). (D) Comparative fluorescence lifetime traces for CDs in both solid and liquid mediums. Figure 4 was reprinted with permission from [178], Copyright 2020 American Chemical Society. This content is not subject to CC BY 4.0.

Novel biomimetic CQDs for the VB_12_ analysis were also synthesized from cytidine diphosphate choline and ethylenediamine (EDA) through a pyrolysis process. A strong luminescence at 450 nm by these CQDs demonstrated ultrasensitive and highly selective detection capabilities for VB_12_ (LOD < 81 nM). In this assay, VB_12_ functioned as the recognition molecule, the biomimetic CQDs acted as the fluorescent indicator, and the observed fluorescence attenuation represented the signal. The fluorescence of the biomimetic CQDs also remained constant across various pH levels, salt concentrations, and under UV light exposure [176]. Dadkhah et al. have recently evaluated VB_12_ concentrations by developing a triple-mode nanosensor based on nicotinamide (NA)-functionalized CQDs through a microwave-assisted hydrothermal process. Linear range and LOD were determined to be 0.1–60 µM and 0.0317 µM, respectively. Interestingly, VB_12_-induced color shifts in NA-CQDs could be captured using a UV–vis spectrophotometer and a custom smartphone app for simultaneous signal reading. When integrated into a smartphone, the NA-CQDs acted as a colorimetric sensor by presenting a reliable linear detection range (4.16–66.6 μM) for VB_12_ with an LOD of 1.40 μM. An RR of 96.52–105.10% with an RSD of 1.32–3.44% for pharmaceutical supplements (i.e., injections and tablets) showed a minimized cross-activity [177]. He et al. synthesized high-yield orange-emitting CQDs using citric acid as precursor in a hydrothermal process. In addition, VB_12_ in tablets, vitamin-infused drinks, and human serum effectively attenuated the CDs’ fluorescence intensity through the IFE, confirming high specificity towards VB_12_ with an LOD of 60.78 nM [178]. Figure 4A shows the clear absorption peak at 530 nm for the CQDs solution, with λ_ex_ = 560 nm and maximum λ_em_ = 520 nm. A vivid orange fluorescence was observed in photos obtained under both daylight and 365 nm UV light exposure. However, there was no dependency between λ_ex_ and λ_em_ of the CDs (λ_ex_ = 360–540 nm; Figure 4B). Figure 4C depicts that, when the solid-state CQDs were exposed to both daylight and 365 nm UV light, they emitted a fluorescence similar to that of CQDs in solution under UV light, with a peak λ_em_ of 595 nm exhibiting a redshift. This indicates the potential application of these CQDs in optoelectronic devices. The fluorescence lifetimes of CQDs in both solid state and aqueous solution were measured, resulting in lifetimes of 3.446 ns and 3.535 ns for the solid state and the aqueous solution, respectively (Figure 4D) [178].

Thermally reduced CDs-based FRET optical sensors were also applied to assess VB_12_ in an aqueous solution within a concentration range from 1 to 12 μg/mL with an LOD of less than 0.1 μg/mL. This approach is highlighted for its simplicity, affordability, sensitivity, and selectivity in detecting biologically important vitamin VB_12_ [179]. Chen et al. [180] in a large-scale produced core–shell CQDs using hydroquinone and EDA as precursors. The rapid formation of the nanocrystalline core was facilitated with the intense exothermic nature of this reaction. Furthermore, these CQDs exhibited green photoluminescence at approximately 525 nm when excited between 320 and 420 nm. A noticeable decrease in the fluorescence intensity was observed after adding VB_12_ to the CQD solution, accompanied by a blueshift in their emission spectrum. The fluorescence intensity ratio at 525 nm of the developed CQDs was proportionally to the VB_12_ level (0.75–100 μM), with an LOD of 0.2 μM. Incremental VB_12_ concentrations significantly diminished the CQDs’ green fluorescence. The FQ of CQDs by VB_12_ demonstrated remarkable specificity, as other studied vitamins and metal ions did not affect their fluorescence. The high selectivity level in detecting VB_12_ led to the development of an easy, visual paper sensor for analyzing VB_12_ fluorescence. Researchers also fabricated a CQD-based fluorescent paper sensor for detecting VB_12_. A strong green fluorescence under UV light was observed when a solution of CQDs was applied to the filter paper. The CQDs’ fluorescence intensity was significantly quenched by increasing the concentration of VB_12_ from 50 to 1000 µM. The authors claimed high specificity of the sensor as FQ through VB_12_ was more noticeable than those through other examined vitamins [180]. In this assay, VB_12_ acted as the recognition analyte, the CQDs functioned as the fluorescent indicator, and fluorescence quenching (observed both in solution and on paper) represented the measurable signal.

There are many studies on CQD-based fluorescent sensing platforms to detect VB_12_ due to their great luminescent potential. Sun et al. synthesized yellow-emitting CDs with a high quantum yield (16.7%) based on ʟ-tartaric acid and urea as precursors in a solvothermal technique. The developed CQDs with excellent photostability and dispersibility could detect VB_12_ in water (LOD of 2.045 μM), manifesting a “turn off” response in their fluorescence. There was a direct linear correlation between the CQDs’ fluorescence intensity and the concentration of VB_12_ (0–200 μM). The IFE-based FQ was identified to be the main mechanism according to detailed microscopic and spectroscopic analyses [181]. The excitation-dependent emission of these yellow-emitting CDs was likely due to the presence of various luminescent centers or emissions arising from surface states. A noticeable shift in color from yellow to red with rising concentration was visible to the naked eye. This trend was also evident in the graphs depicting the ethanol solutions of CQDs at different doses from 2.5 to 150 μM under 450 nm excitation [181]. In this sensing system, VB_12_ was the recognition analyte, the yellow-emitting CQDs acted as the fluorescent indicator, and fluorescence quenching (“turn off” response) was recorded as the signal. As well, Zhao et al. synthesized bright yellow fluorescent nitrogen-doped CDs (N-CQDs) with a quantum yield of 32% via a hydrothermal process with *O*-phenylenediamine and 4-aminobenzoic acid as precursors. They stated that when these QDs were excited at 390 nm, a pronounced decrease in their fluorescence intensity at 567 nm was observed upon interaction with VB_12_. A fluorescence sensor based on these QDs was developed in an aqueous medium, indicating a linear response to two different concentration ranges of VB_12_ (0 to 90 μM and 140 to 250 μM), with a low LOD of 0.119 μM [182]. Another hydrothermal process in the presence of citric acid and diethylenetriamine (DETA), respectively, as carbon and nitrogen sources, was performed to prepare blue-emitting N-CQDs (λ_ex_ = 391 nm and λ_em_ = 438 nm) with a size of 2.3 nm and an exceptional quantum yield of 58%. The presence of Co^2+^ ions in water with very low cross-reactivity with other metal ions could quench the fluorescence of the developed CDs, confirming the high sensor selectivity. A linear range of 0–90 μM and a LOD of less than 0.4 μM were recorded to monitor these metal ions, which can be particularly used to track the presence of VB_12_ or Co^2+^-based medications [183]. Zhang et al. reported the synthesis of onion-derived CQDs via a hydrothermal technique and their subsequent functionalization with PEI. The findings revealed that the 2.82 nm PEI-CQDs with hydroxy and amino groups on their surface exhibited excitation and emission peaks at 340 nm and 462 nm, respectively, with a fluorescence quantum yield of 8.68%. The fluorescence of PEI-CQDs was selectively quenched by Co^2+^ ions, making them effective for the detection and quantification of Co^2+^ in various samples such as VB_12_. The LOD and linear range for detecting Co^2+^ were determined to be 0.048 μM and 0.05–11 μM, respectively. An RR of 97.0–100.64% showed that PEI-CQDs could be effectively utilized in monitoring Co^2+^ levels in tap water [184].

#### Nitrogen-doped carbon quantum dot-based fluorescent sensors

N-CQDs are particularly practical for fluorescent sensing of VB_12_, owing to their synergistic properties, including enhanced fluorescence, precise target interaction, high quantum yield, biocompatibility, stability, and cost-effective production. The integration of nitrogen atoms not only increases their fluorescence intensity but also reinforces targeted interactions with Co^2+^ and VB_12_, revealing significant changes in fluorescence signals. These modifications are based on the nitrogen contribution to the dots’ quantum yield, ensuring the detection of very low concentrations of Co^2+^ and VB_12_. Their compatibility with biological samples allows for their use without affecting detection quality or creating health risks. Furthermore, the resilience of N-CQDs to different environmental conditions ensures reliable sensing across diverse sample matrices. Coupled with their economical and simple synthesis process, N-CQDs would be potent and promising nanomaterials for the sensitive and selective detection of Co^2+^ and VB_12_, with widely ranging implications for healthcare diagnostics and nutritional analysis [185–186].

Bano et al. synthesized fluorescent N-CQDs with a high quantum yield of 57%. These nanomaterials showed a robust blue emission. These N-CQDs also demonstrated resilience to high salt concentrations and maintained their photostability for over six months. Utilizing N-CQDs as a sensor for the selective “turn off” detection of Co^2+^ proved an LOD of lower than 0.12 μM within a linear range of 0.5–3.0 μM. Different mechanisms were involved in detecting Co^2+^, including IFE, static quenching effect (SQE), aggregation, and the formation of complexes with the N-CQDs’ amino groups. The fluorescence quenched by Co^2+^ could be “turned on” again by adding chemical compounds such as glutathione, ascorbic acid, cysteine, and ethylenediaminetetraacetic acid (EDTA), proving the N-CQDs’ potential as a reversible probe for Co^2+^ determination. The standard recovery test of Co^2+^ spiked into VB_12_ samples showed that N-CQDs were an efficient detection tool with 96.0–105.5% RR and 1.34–2.54% RSD [185].

Kalaiyarasan and Joseph assessed VB_12_ through pH-dependent (2.0–9.0) FRET quenching of N-CQDs prepared by a hydrothermal process (160 °C, 6 h) in the presence of TSA and DETA. N-CQDs had a polycrystalline structure under acidic conditions, whereas a mixed crystalline structure was observed for N-CQDs in basic media. The authors also indicated that VB_12_ could strongly quench the fluorescence of the developed N-CQDs through the FRET mechanism. High-resolution transmission electron microscopy analysis showed CQDs with diameters of 2.1–3.5 nm embedded in an amorphous carbon matrix, while dynamic light scattering indicated a dominant size of 10.8 nm. Aggregated particles were likely due to nitrogen-induced hygroscopic effects. Lattice spacings of 0.229, 0.334, and 0.186 nm matched the (100), (006), and (105) planes of graphite. The XRD pattern showed a broad peak at 25°, consistent with disordered carbon and the (006) graphite plane. The LOD and linear range of VB_12_ were ≈210 pM and 1 nM–20 μM, respectively [186]. Nandi et al. [187] and Yu et al. [188] also produced fluorescent NCQDs with high optical stability from different precursors via hydrothermal synthesis (Table 2). Compared to Yu et al.’s work, Nandi et al. obtained a higher quantum yield (22.7% vs 15.9%; Table 2). Nandi et al. reported that the developed NCQDs had good potential to measure bilirubin and VB_12_ through FRET- and IFE-based FQ, respectively. The efficacy of this kind of fluorescent nanosensors (λ_ex_ = 360 nm, λ_em_ = 450 nm) was confirmed in real samples such as VB_12_ tablets, human serum, and energy drinks with an RR of 95.06–112.48% [187]. Yu et al. found that NCQDs prepared from the precursors ʟ-tartaric acid, urea, and dimethylformamide could detect VB_12_ with an LOD of 2.101 μM; also, they maintained their photostability under diverse pH levels, temperatures, and ionic strengths [188].

#### Nitrogen and sulfur co-doped carbon quantum dot-based fluorescent sensors

Nitrogen and sulfur co-doped CQDs (N,S-CQDs) present a remarkable advancement in the field of molecular sensing due to their enhanced properties over quantum dots doped solely with nitrogen. The combined doping with nitrogen and sulfur not only increases the photoluminescence of the CQDs for brighter and more stable fluorescence but also improves their sensitivity and selectivity towards target molecules. This can be attributed to the synergistic effect of nitrogen and sulfur doping, which introduces more active sites and facilitates selective binding to specific molecules (such as VB_12_). Moreover, N,S-CQDs demonstrate superior chemical and photostability, broadening their application beyond sensing to bioimaging, drug delivery, and photocatalysis [189].

Li et al. synthesized N,S-CQDs with a quantum yield of 10.4% from thiamine nitrate via a hydrothermal process. VB_12_ and the synthetic lemon-yellow azo dye tartrazine (acceptor) could well quench N,S-CQDs’ fluorescence (donor) via FRET. Energy transfer rate and efficiency between donor and acceptor compounds were increased by increasing acceptor concentrations and varied with different excitation wavelengths (338–408 nm). VB_12_ and tartrazine were selectively and sensitively detected using the N,S-CQD-based probe with LODs of 0.0156 and 0.018 μM, respectively. High RRs of 97.5–104.2% for VB_12_ (RSD = 0.11–2.08%) and 91.0–110.6% for tartrazine (RSD = 0.09–3.35%) in milk and two types of vitamin drinks demonstrated the sensor’s performance [190]. Orange-emitting N,S-CQDs with good stability and quantum yield were also developed for label-free monitoring of VB_12_ through a hydrothermal reaction between the precursors *o*-phenylenediamine and thiourea. The nanomaterials exhibited a unique photoluminescent signature, with emission peaks at 420 nm and 565 nm, remaining constant regardless of the excitation wavelength. This feature enabled the identification of VB_12_ based on IFE, as its absorption spectrum extensively overlaps with the emission peaks of the nanomaterials. Under optimal reaction conditions (at 25 °C in a pH 6.0 methylethanesulfonate buffer for 5 min), there was a linear relationship between the VB_12_ concentration and the fluorescence quenching ratio at 565 nm (0.25–20 μM), with an LOD of 77.5 nM [191]. In this sensing model, VB_12_ functioned as the recognition analyte, the N,S-CQDs acted as the fluorescent indicator, and the quenching of orange emission provided the detectable signal.

Similar nanomaterials based on ʟ-cysteine as precursor were prepared for the selective and visual determination of Co^2+^ ions within a linear range of 1–50 μM and an LOD of 0.026 μM [192]. Sun et al. found that the addition of H_2_O_2_ or EDTA can restore (turn on) the fluorescence of N,S-CDs that was previously quenched (turned off) by Co^2+^ ions. The quenching was attributed to an SQE mechanism, which involves electron transfer through the creation of a complex between Co^2+^ and the functional groups present on the N,S-CDs [192]. Luo and Jiang earlier explained that the robust oxidizing capabilities of H_2_O_2_ could make the fluorescence system based on N,S-CDs a promising “on–off–on” fluorescent switch sensor for the detection of H_2_O_2_ [193]. The fluorescence of the combination of N,S-CDs, and Co^2+^ was also rejuvenated with EDTA. This chemical, due to its strong chelating ability, can simply form stable complexes with metal ions and does not alter the fluorescence intensity of N,S-CDs. Thus, the presence of EDTA reduces the impact of Co^2+^ on the N,S-CDs, facilitating the recovery of the quenched fluorescence [194]. In a similar procedure, Mohammadi et al. hydrothermally synthesized N,S-CQDs by utilizing pomegranate juice as the carbon source and cystamine for both sulfur and nitrogen in order to sensitively detect VB_12_ in the concentration range of 0–110 μM with an LOD of 0.082 μM. To demonstrate the practicality of this approach, a VB_12_ ampoule was analyzed as a real-world sample. The obtained high RR confirmed the effectiveness and applicability of N,S-CQDs for pharmaceutical and biological purposes [195].

#### Nitrogen and phosphorus co-doped carbon quantum dot-based fluorescent sensors

Nitrogen and phosphorus co-doped CQDs (N,P-CQDs) represent a significant advancement in the field of nanomaterial-based sensing, offering enhanced capabilities compared to their N-CQDs. By introducing both nitrogen and phosphorus into the carbon lattice, N,P-CQDs exhibit modified electronic structures and surface chemistries, leading to excellent photoluminescence properties that can be finely tuned for specific sensing applications [196]. This co-doping approach not only enhances the sensitivity and selectivity of these QCDs towards various analytes but also improves their solubility and stability across different environments. The presence of nitrogen and phosphorus allows for the introduction of diverse functional groups on the CQDs’ surface, facilitating specific interactions with target molecules. These interactions can induce changes in the QDs’ luminescence, serving as a basis for detecting a wide range of substances [197].

Wen et al. hydrothermally prepared N,P-CQDs with a 64% yield using a blend of frozen tofu, EDA, and phosphoric acid. This N,P-CQDs-based sensor could selectively and sensitively detect Co^2+^ ions with an LOD of 0.058 μM by fluorescence quenching [198]. Similar to the work of Sun et al. [192] on N,S-CDs-based fluorescent sensors in detecting Co^2+^ ions, Wen et al. reported that the fluorescence quenching can be reversed by adding EDTA. Accordingly, the LOD of 0.098 μM for the concentration of EDTA in solutions was determined. This dual functionality establishes that these CQDs as an effective “off–on” fluorescence sensor for both Co^2+^ ions and EDTA. The practical application of this method was validated by accurately measuring Co^2+^ levels in tap water (96.3–103.3% RR, 1.78–3.03% RSD) and EDTA in contact lens solutions (98.6–104.3% RR, 1.87–2.64% RSD) [198]. Zhang et al. also designed a new N,P-CQDs with good water solubility and favorable biocompatibility, which were hydrothermally synthesized using a mixture of sucrose (C source), 85% phosphoric acid (P source), and 1,2-EDA (N source). The excitation/emission peaks are at 365/451 nm, and bright blue, green, or red emission was found depending on whether the excitation wavelengths of the laser were set to 408, 488, or 543 nm, respectively. The authors found that the fluorescence could be quenched by both VB_12_ and Co^2+^ ions by a combination of SQE and IFE mechanisms. The linear range for the VB_12_ concentration was assessed to be 2.0–31 μM, whereas the linear response to Co^2+^ ions occurred in two ranges of 1.7–12 μM and 28–141 μM. The LOD of VB_12_ and Co^2+^ were 3.0 nM and 29.4 nM, respectively. In this sensing model, VB_12_ or Co^2+^ served as the recognition analytes, the N,P-CQDs acted as the fluorescent indicators, and the quenching of multicolor emissions provided the measurable signals. To validate the sensor’s performance, the nanoprobe was successfully applied to analyze VB_12_ (96–108% RR and 2.7–8.1%) and Co^2+^ (112–132% RR and <3.0%) in tablets/injections and spiked water samples, respectively [199]. In another study done by Wang et al., N,P-CDs with an 18.38% quantum yield as well as excellent water solubility and luminescent qualities were successfully synthesized through a one-step hydrothermal method using ʟ-arginine and phosphoric acid as precursors [200]. They pointed out that the optimal emission could be observed at 444 nm upon excitation at 340 nm. The developed N,P-CDs could efficiently and selectively monitor VB_12_ via the IFE mechanism. This highly fluorescent sensor responded to VB_12_ concentrations within two linear ranges of 1.99–98.6 μM and 98.6–176 μM, with a very low LOD of 0.059 μM. Moreover, the sensor’s efficacy was affirmed by analyzing VB_12_ in various vitamin tablet formulations (99.6–109.0% RR and 0.66–1.06%) and blood serum (82.1–102.1% RR and 0.37–4.66%) samples [200].

#### Fluorescent sensors based on other chemically doped quantum dots

Cadmium sulfide (CdS) and cadmium telluride (CdTe) QDs have unique optical properties and applications. CdS QDs are primarily utilized in optoelectronics and photocatalysis, benefiting from their strong UV–visible absorption and photoluminescence. In contrast, CdTe QDs are favored in medical imaging and biosensing, owing to their tunable emission across a broader visible to near-infrared spectrum, which is particularly advantageous for biological applications requiring deep tissue penetration. While both types serve in sensing technologies, their inherent differences in emission range and stability make each suitable for specific tasks, with CdTe QDs being especially valuable in the food and pharmaceutical industries for sensitive and selective detection of metabolites and molecules, such as VB_12_ analysis [201–202]. Gore et al. fabricated an innovative FRET probe to measure VB_12_ in aqueous environments through mercaptopropionic acid (MPA)-functionalized CdS QDs, made from cadmium chloride and sodium sulfide. In the range of 5.0–100 μg/mL, there was a linear relation between CdS QDs’ fluorescence intensity and VB_12_ level, while an LOD of 6.91 μg/mL was recorded. High selectivity was determined for this fluorescent sensor in the presence of many interfering substances such as metal ions (e.g., K^+^, Ca^2+^, and Mg^2+^), ammonium cations (NH^4+^), sugars (e.g., starch, maltose, glucose, fructose, sucrose, lactose, and dextrose), ascorbic acid, tyrosine, urea, and thiourea. In this platform, VB_12_ functioned as the recognition analyte, MPA-functionalized CdS QDs served as the fluorescent indicator, and the FRET-induced fluorescence quenching provided the sensing signal. The MPA-functionalized CdS QDs could be efficiently applied to monitor VB_12_ in blood serum, urine, and multivitamin injections without any sample pretreatment (97.15–99.49% RR, 0.63–2.16% RSD) [201]. Shamsipur et al. developed a selective and sensitive IFE-based method using thioglycolic acid (TGA)-capped CdTe QDs in order to monitor VB_12_ in aqueous media. The fluorescence quenching rate of the prepared TGA-CdTe QDs (λ_ex_ = 390 nm and λ_em_ = 523 nm) under optimum conditions had a linear correlation with VB_12_ dose within concentration ranges of 0.02–0.4 and 1.5–70.0 µM VB_12_ with an LOD of 0.002 μM. The sensor potential was confirmed by determining VB_12_ in pharmaceutical injections (95–105% RR, 2.4–4.5% RSD) [202]. Wang et al. developed fluorescence sensors based on 5.36 nm sulfur quantum dots (SQDs) to detect Co^2+^ ions. They introduced a novel assay leveraging aggregation-caused quenching of SQDs for detecting these ions with a linear concentration range of 0–9 µM, with an LOD of 0.02 µM [203]. A new fluorescent sensor was also synthesized based on Mn^2+^-doped ZnS QDs, using zinc acetate dihydrate, sodium sulfide flakes, and manganese acetate tetrahydrate as precursors, through an integrated thermal and mechanical treatment [204]. Jia et al. hydrothermally produced 3.3 nm boron-doped carbon dots (BCDs) using phenylboronic acid as the precursor. These nanomaterials with a quantum yield of 12% exhibited excitation and emission wavelengths of 247 nm and 323 nm, respectively. The fluorescence of BCDs could be quenched by sorbate and VB_12_ through IFE and FRET mechanisms. Linear range and LOD of VB_12_ were determined to be 0.20–30 μM and 0.008 μM, respectively [8].

#### Graphene quantum dot-based fluorescent sensors

This type of sensor is considered an innovative approach to VB_12_ detection due to the exceptional surface area, conductivity, and biocompatibility of graphene and GO. These characteristics facilitate rapid, reliable assays, critical for monitoring VB_12_ levels in complex matrices and promoting the quality control and efficacy of dietary supplements and fortified foods [205]. A new fluorescent probe, 1,8-diaminonaphthalene (DAN)-functionalized GQDs (DAN-GQDs), designed by Ravi et al. [206], could sensitively detect negligible levels of vitamin B_9_ (LOD = 1.73 × 10^−15^ M) and MeCbl (LOD = 6.37 × 10^−12^ M) in water using absorption and fluorescence methods. This technique utilized IFE due to the overlap between the absorption spectra of vitamin B_9_ and VB_12_ and the emission spectrum of DAN-GQDs. The derived association constant values indicated that DAN–GQD interacts with vitamin B_9_ and MeCbl at stoichiometric ratios of 1:2 and 1:1, respectively. Investigation into the time-resolved fluorescence decay patterns (λ_ex_ = 328 nm, λ_em_ = 338 nm) validated that the reduction in fluorescence intensity of DAN–GQD, triggered by the addition of vitamin B_9_ and MeCbl, is a consequence of the non-radiative dissipation of energy from excited electron states. In this biosensing setup, MeCbl served as the recognition analyte, DAN–GQDs acted as the fluorescent indicator, and the IFE fluorescence quenching constituted the sensing signal. They also reported that the DAN-GQDs-based sensor revealed a desirable performance with commercial FA and MeCbl in water [206]. Co-doping GQDs with heteroatoms (e.g., nitrogen or sulfur) enhances their electronic and optical properties, enhancing fluorescence efficiency for better performance in sensing, bioimaging, and optoelectronics. This approach modifies the GQDs’ bandgap, increases PL yield, and improves stability and surface functionality, enabling more selective and sensitive interactions in applications like sensors and catalysis [207–209]. Boonta et al. assessed the fluorescence detection of Co^2+^ ions in water via synthesized nitrogen and sulfur co-doped GQDs (N,S-GQDs). The prepared N,S-GQDs could be quenched by Co^2+^ through interactions between the metal ions and the surface functional groups of the fluorescent probe. Furthermore, the aggregation of N,S-GQDs was induced by the addition of Co^2+^, leading to an enhancement of UV–vis absorption at 430 nm and a color transition to yellow-brown within 3 min. Linear range and LOD for Co^2+^ were determined to be 0–40 μM and 1.25 μM, respectively. Also, the authors successfully evaluated the fluorescent sensor probe’s potential to measure Co^2+^ in real water samples [208]. Martins et al. monitored multivitamins (LOD_VB12_ = 0.32 nM) in classic and fruit-based energy drinks using electrodes functionalized with N,S-GQDs [209].

#### Carbonized polymer dot-based fluorescent sensors

Gao et al. prepared CPDs using a facile, one-pot method with tetrachlorobenzoquinone and EDA as precursors, catalyzed by a Schiff base condensation reaction. These CPDs are characterized by their unique dual-emission properties, with blue emission at 445 nm and yellow emission at 575 nm, demonstrating exceptional photostability and antioxidant capabilities. The authors also developed a ratiometric fluorescent nanoprobe for the accurate detection of VB_12_, achieving a sensitive assay within a linear range of 0.25–100 μM and a very low LOD of 0.14 μM. The researchers claimed that the applied innovation not only simplified the CPDs fabrication process with lower cost and complexity but also signified a significant step forward in the field of biosensing, offering a potent tool for the design of advanced fluorescent nanoprobes for a wide array of analytical and biomedical applications [210].

#### Silicon quantum dot-based fluorescent sensors

Silicon-based nanomaterials, including nanoparticles, nanowires, and nanorods, due to their negligible toxicity and inherent biocompatibility, can be utilized for biomedical applications. The unique optical properties of these materials, along with their non-toxic nature and controllable surface functionalization, have rendered them intriguing substances for fabricating advanced fluorescent sensors [211]. Silicon quantum dots (SiQDs) as zero-dimensional fluorescent silicon nanomaterials have acceptable water solubility for biological applications such as photoluminescent sensing and bioimaging of different ions and biomarkers [212].

Long et al. designed label-free SiQD-based fluorescent sensors using microwave-assisted synthesis under normal pressure to detect VB_12_. This micronutrient, owing to the IFE, could quench the SiQDs’ fluorescence. The authors applied quercetin and doxorubicin as controls thanks to the alignment of their absorption peaks with SiQDs’ excitation or emission peaks, which aided in elucidating the quenching mechanism. The quenching efficiency was found to depend on the overlap extent between the quencher’s absorption peak and the SiQDs’ excitation or emission peaks, with a greater overlap leading to increased quenching efficiency. A linear increase in fluorescence quenching efficiency was observed within a VB_12_ concentration range from 0.5 to 16 μM, while an LOD of 0.158 μM was obtained. In this sensing system, VB_12_ acted as the recognition analyte, SiQDs were the fluorescent indicator, and fluorescence quenching via the IFE mechanism provided the signal. Furthermore, the performance of SiQDs-based fluorescent sensors for VB_12_ quantification was affirmed by evaluating a RR between 97.7% and 101.1% in tablets and human urine samples [213]. In another research, green luminescent SiQDs were used for the assessment of VB_12_ and as antibacterial agent [214]. Zhao et al. synthesized them using thiourea and catechol via a microwave-assisted hydrothermal process. The developed SiQDs showed a linear range of 0.05–30 μM and an LOD of 0.05 μM for VB_12_. The authors also realized that these nanomaterials had good antimicrobial activity against *Staphylococcus aureus* with a minimum inhibitory concentration of 250 μg/mL. However, they pointed out that at least 1.3 mg/mL is needed to inhibit the biofilm growth of this bacterium. The SiQDs–lysozyme complex effectively inactivated *S. aureus*, achieving a low MIC of 10 μg/mL and hindering biofilm growth at 62.5 μg/mL, thanks to singlet oxygen, charge effects, and peptidoglycan hydrolysis [214]. In general, the antibacterial mechanism of SiQDs can be due to (i) their small particle size, facilitating easy penetration through bacterial walls and membranes, thus inducing bacterial death, (ii) electrostatic interactions, where SiQDs disrupt membrane structures via electrostatic adsorption, leading to bacterial death, and (iii) the generation of reactive oxygen species, which can damage microbial external membranes and proteins, ultimately causing bacterial death [215–220]. *S. aureus* displayed bright orange, green, and blue fluorescence when excited by lasers at 557, 493, and 353 nm, respectively. The visible distinct spherical structure of *S. aureus* suggested the bacteria cells successfully absorbed the SiQDs [214]. Sullam et al. developed a new fluorescence probe based on SiQDs to measure Co^2+^ in a linear range of 1–120 µM and an LOD of 0.37 µM. Highly water-soluble and stable SiQDs were hydrothermally produced by mixing 3(2-aminoethylamino)propyldimethoxymethylsilane and poly(vinylpyrrolidone) for detecting Co^2+^ through static quenching. The authors also developed a cheap SiQD-based test paper with excellent selectivity and sensitivity and minimized cross-activity, and successfully utilized it to quantify Co^2+^ levels in natural water samples. The FQ was notably intensified by raising the concentration of Co^2+^, aligning closely with the outcomes obtained in the absence of interfering ions [221].

#### Fluorescent sensing with natural carbon-based quantum dots

The use of fluorescent sensing with natural carbon-based quantum dots (nCQDs) is crucial not only for ensuring food safety and quality but also for the pharmaceutical industry, as it provides a highly sensitive, non-toxic, and eco-friendly method for detecting contaminants, nutrients, and active pharmaceutical ingredients. This innovative approach enables the rapid, accurate monitoring and quality control of food products and pharmaceuticals, which is essential for preventing health hazards, adhering to rigorous regulatory standards, and enhancing consumer preference in both the food supply chain and pharmaceutical products [29,222–224].

Recently, a lot of natural resources have been used to synthesize CQDs as cost-effective and eco-friendly sensing materials by carbonation and hydrothermal methods. Preethi et al. obtained a fluorescent biosensor via ultrasound-assisted green synthesis of CQDs from curry berries (*Murrayakoenigii* L.) for VB_12_ quantification (LOD = 0.04 μM). The fluorescent intensity was progressively diminished in the presence of VB_12_ because a significant reduction in this parameter was observed from 0 to 0.40 μM. In this biosensing design, VB_12_ was the recognition molecule, CQDs acted as the fluorescent indicator, and the fluorescence attenuation due to coordination with Co^2+^ ions served as the measurable signal [222]. The presence of oxygen-containing groups on the CQDs’ surface allowed for the formation of complexes with Co^2+^ ions via coordination interaction, facilitating electron transition from the CQDs to the Co^2+^ ions [225]. The complex formed between CQDs and Co^2+^ ions resulted in a substantial reduction in the CQDs’ fluorescence. Researchers also confirmed the sensor efficiency by calculating the RR (93.0–97.6%) in milk powders at pH 7.0 with three concentrations of 0.1, 0.2, and 0.3 µM VB_12_ [222].

Xiang et al. developed innovative nCQDs obtained from the edible mushroom *Lactarius hatsudake* as fluorescent sensors for detecting VB_12_. In a hydrothermal process at 200 °C for 12 h, they successfully synthesized new 3 nm nCQDs with a quantum yield of 22.88% to detect VB_12_ concentrations within a linear range of 0–20 µM and an LOD of 36.9 μM. They evaluated the selective performance of nCQDs was validated by analyzing the content of VB_12_ in three different milk samples with an RR of 91.50–100.40% and an RSD as low as 2.08% [226]. A comparison in the RR and RSD of these nCQDs with other analytical methods, such as HPLC-ICP-MS (81.3–103.6% RR and 0.4–4.6% RSD) [227] and HPLC-MS/MS (75.2–89.5% RR and 3.6–5.9% RSD) [228], showed acceptable performance of the sensors based on *L. hatsudake-*derived CQDs. Yu et al. also synthesized N-doped nCQDs for sensing pH and VB_12_. The precursor and nitrogen source were *Saccharomyces* and ethanediamine, respectively. The fluorescence intensity and lifetime of N-doped nCQDs were increased by shifting the pH value from 14 to 2. Furthermore, the fluorescence intensity revealed a highly reversible capability from pH 13 to 2, maintaining its strength without a meaningful decrease after ten consecutive cycles. These sensors, according to the FRET mechanism, could also detect VB_12_ with an LOD of 2.19 μM [229]. Likewise, Zhao et al. prepared new N-doped nCQDs for Co^2+^ ions and pH sensing based on the precursors of seaweed kelp and EDA under 800 W microwave irradiation at 200 °C for 10 min. The luminescence of CDs displayed a consistent linear correlation across a pH spectrum of 3 to 8 and was utilized for visually identifying Co^2+^ ions. They mentioned that adding Co^2+^ visibly changed the solution’s color from clear to brownish-yellow under normal light and from bright blue to dark blue under UV light. Linear range and LOD of Co^2+^ concentration were 1–200 μM and 0.39 μM, respectively. This fluorescent sensor was effectively used to monitor these ions in samples of water collected from a river [230]. Another study on the hydrothermal synthesis of N-doped nCQDs using biomass quinoa saponin powders and EDA was performed. A high quantum yield (22.2%) with a small particle size (≈2.25 nm) was recorded for these fluorescent materials, which showed excitation wavelength-dependent blue light emission. The developed N-doped nCQDs detected Co^2+^ ions within the linear range of 20–150 μM with an LOD of 0.49 µM [231]. Ji et al. also synthesized bright-blue photoluminescent CDs from the bacterium *Weissella* sp. Kl-3 through a hydrothermal technique. CDs sensitized by ampicillin sodium with multicolor fluorescence emission characteristics were efficiently utilized as a fluorescence nanosensor to detect Cr^6+^ and VB_12_ via the IFE mechanism. Linear range and LOD for Cr^6+^ and VB_12_ concentrations were determined to be 0–50 μM and 0–25 μM, as well as 0.10657 μM and 0.0515 μM, respectively. This nanosensor was able to detect Cr^6+^ ions in water and VB_12_ in serum and milk samples [232]. In a recent research by Chen et al., nCQDs using edible bird’s nest, a traditional Chinese delicacy, and distilled water have been hydrothermally prepared to analyze VB_12_ in serum [233]. These nanomaterials represented excellent features such as high water solubility, fluorescence efficacy, biocompatibility, and stability across a wide pH spectrum (3.0–11.0) and in solutions of high ionic strength. The interaction of nCQDs with VB_12_ led to a remarkable reduction in the fluorescence intensity of nCQDs with increasing VB_12_ doses. Linear range and LOD were 0–100 µM and 0.24 µM, respectively. An RR of 96.2–100.3% of VB_12_ in human serum revealed that the developed nCQDs can be used for clinical applications [233]. Tiwari et al. fabricated CDs derived from *Cannabis sativa*, co-doped with nitrogen (10.71%) and sulfur (1.94%; N–S@CsCD), which were able to serve as highly efficient nanosensing platforms for temperature and VB_12_. These 4–6 nm CDs showed an excitation-independent emission with a peak λ_em_ at ≈414 nm. N–S@CsCDs maintained excellent stability over time and at different salt and pH levels. Besides, N–S@CsCD in the presence of VB_12_ exhibited selective FQ, achieving an LOD of 7.87 μg/mL. The unchanged fluorescence lifetimes of N–S@CsCD in the absence and presence of VB_12_ indicated a static mechanism for the sensing behavior. Eventually, the selectivity of these sensors was determined by assessing the content of VB_12_ in pharmaceutical injections. RR and RSD values were 93.75–98.20% and 0.28–1.09%, respectively [234].

**Figure 5:**
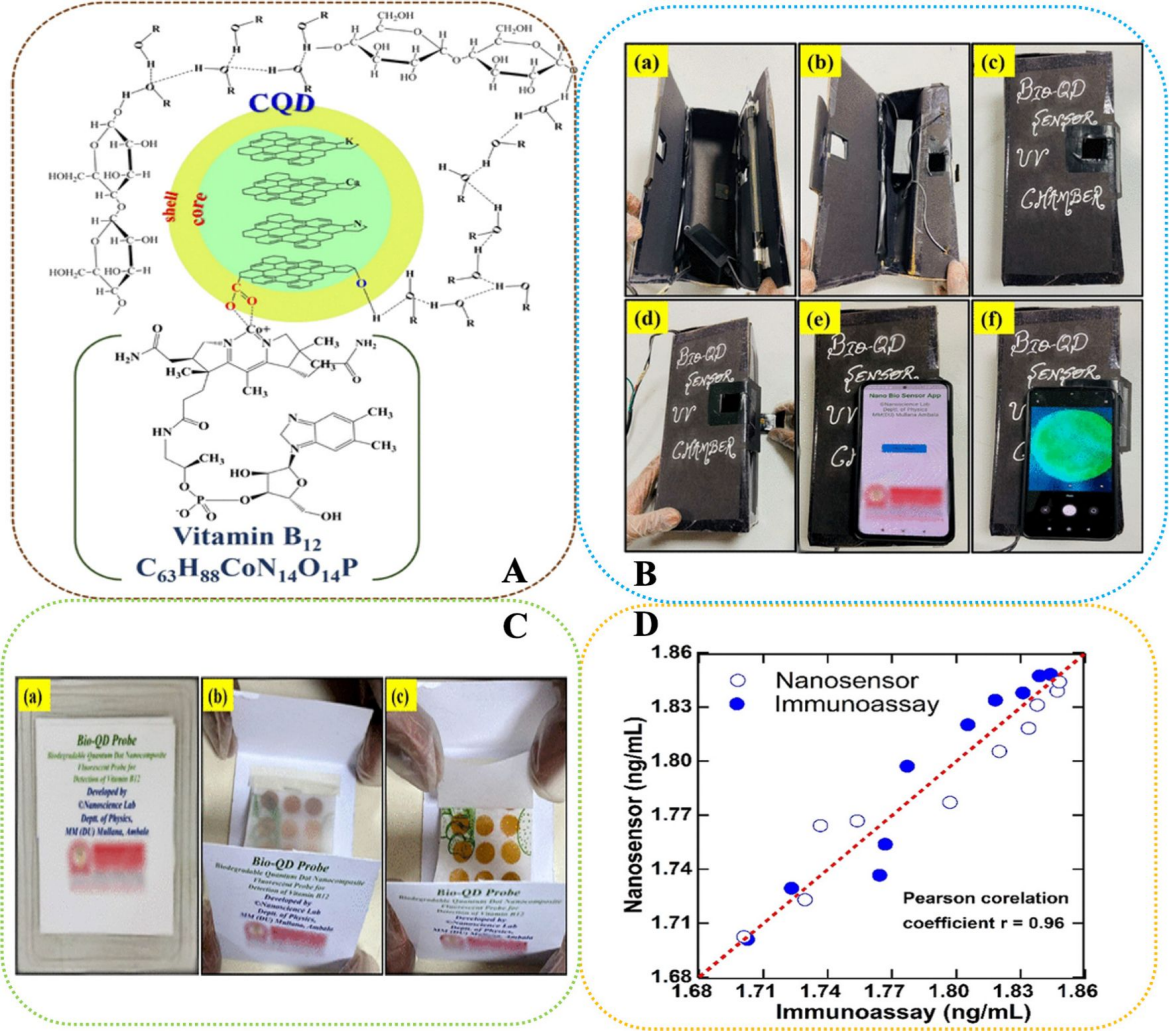
(A) Potential pathways for the reduction in fluorescence of VB_12_ within a bioplastic polymer matrix. (B) Proof-of-concept testing setups for CQDs in bioplastic nanocomposite-based nanosensor with real samples for affordable POC uses. (a–c) Digital images of tailored UV chamber for sample analysis, including UVC tube (11 W, 254 nm) assembly and adapter circuit configuration. (d) Procedure for loading samples and (e, f) RGB (red, green, and blue) format image acquisition via specialized smartphone applications. (C) Digital photographs of (a–c) customized packaging of *A. marmelos*-derived CQDs in bioplastic nanocomposite-based fluorescence nanosensors for low-cost POC applications, and (D) Pearson’s correlation analysis of bioplastic nanocomposite nanosensor versus standard reference method for VB_12_ detection. Figure 5 was adapted with permission from [235], Copyright 2024 American Chemical Society. This content is not subject to CC BY 4.0.

Kansay et al. fabricated a novel solid-state fluorescence nanosensor based on CQDs obtained from *Aegle marmelos* fruit extract and doped with N, K, and Ca, integrated into a bioplastic nanocomposite, enabling the precise and sensitive quantification of VB_12_. The nanosensor facilitated dual on-site VB_12_ monitoring through fluorescence and a visible color change, captured and analyzed by a smartphone camera using a custom Android app for real-time, quantitative feedback. Linear range and LOD of VB_12_ were obtained to be 0.01–100 µM and 0.00916 µM, respectively. A combined process involving adsorption and FRET between VB_12_ and the CQDs was the main fluorescence quenching mechanism (Figure 5A) [235]. Similar quenching phenomena have been reported in studies using a variety of luminescent QD-based nanoprobes for VB_12_ detection [8,123,176,186,204]. Figure 5B illustrates cost-effective and simple tools for detecting VB_12_ through fluorescence in POC scenarios. Digital shots display a custom protective enclosure for a conventional ultraviolet C (UVC) light tube (11 W, 254 nm), equipped with a compartment for inserting samples and a viewing hole to utilize a smartphone’s rear camera. Once the test sample is positioned within the UV chamber, a digital image can be captured using specialized Android smartphone applications. These apps proceed to analyze the image in RGB (red, green, and blue) format and subsequently report the VB_12_ concentration in the sample. Figure 5C illustrates an economical packaging approach for the novel nanosensors, further increasing their applicability in POC settings. The effectiveness of the newly created nanosensor compared to a standard reference method (i.e., immunoassay technique based on electrochemiluminescence (ECL) technology) was examined by employing Pearson’s correlation test (*r* = 0.96; Figure 5D). The authors could successfully apply these nanomaterials to assess VB_12_ contents in commercial beverages and pharmaceutical supplements (98.6–106.4% RR). This proposed that the newly developed fluorescent nanosensor, which utilized CQDs embedded in bioplastic nanocomposites, was effective for the quantitative analysis of VB_12_ [235].

### VB_12_-specific quantum dot fluorescent sensors for cell-bioimaging applications

The genetically encoded fluorescent nanosensor (SenVitAL) has been successfully utilized to measure the intracellular VB_12_ content in *E. coli* cells under in vivo conditions. Exposing *E. coli* cells to VB_12_ led to a notable enhancement in FRET efficiency [125]. Bogner et al. [236] and Zhang et al. [237] earlier found that genetically encoded FRET-based sensors could be applied to evaluate the kinetics of the accumulation of plant/microbial metabolites in the cells. Ahmad et al. reported that the SenVitAL sensor measured VB_12_ concentrations within the cytosol of yeast and mammalian cells, demonstrating its non-destructive real-time monitoring potential of this vitamin in eukaryotic systems. Therefore, this sensor emerged as a novel tool to scrutinize VB_12_ import and metabolism, potentially aiding in exploring their intricate roles in biological processes. The sensor’s in vivo specificity was confirmed by adding ascorbic acid and vitamin B_1_ to the bacterial cells expressing the sensor for 35 min. The addition of these metabolites did not lead to any notable shifts in the FRET ratio over this period, as compared to the initial FRET ratio measurements at 0 min. Nevertheless, a significant alteration in the FRET ratio was only detected in the presence of VB_12_ [125].

Meng et al. [173] initially assessed the toxicity of orange-emitting CDs using the MTT (3-(4,5-dimethylthiazol-2-yl)-2,5-diphenyltetrazolium bromide) assay on the PC-12 cell line (derived from a rat’s adrenal medulla tumor or pheochromocytoma) and found that these cells had a survival rate of above 80% after exposure to various concentrations of the CDs (0–300 μg/mL), confirming their suitability for applications in bioimaging. Here, VB_12_ functioned as the recognition molecule, the CDs acted as fluorescent indicators, and the gradual attenuation of cytoplasmic orange fluorescence represented the measurable signal. The authors then evaluated the potential of orange-emitting CDs in tracking VB_12_ in live cells. Incubating PC-12 cells with 0.25 mg/mL CDs for 30 min resulted in a pronounced orange fluorescence within the cytoplasm, indicating successful internalization and dispersion of CDs throughout the cytoplasmic area. Upon adding VB_12_ to the PC-12 cells’ nutrient solution, a gradual decrease in orange fluorescence was observed, confirming the ability of CDs to monitor the presence of VB_12_ in living cells [173].

Wang et al. also employed the standard MTT assay to investigate the cytotoxicity of biomimetic CQDs on the human cervical cancer cell line HeLa before their biological application. Results showed that the cell viability exceeded 85% after a two-day incubation with these CQDs at 100 μg/mL. Green and red fluorescence of the biomimetic CQDs was measured after excitation at wavelengths of 488 and 543 nm, respectively. The merged images, appearing gold, combine both green and red signals. Cells were first incubated with the CQDs for 4 h for 2D imaging. The 3D images demonstrated precise imaging of HeLa cells, with these CQDs visibly entering the cytoplasm and nucleus, confirming their internalization within the cells, rather than superficial attachment. To assess the intracellular colocalization of CQDs and VB_12_, cells in 3D culture pre-treated with CQDs were subsequently incubated with VB_12_ for 5 h and then imaged. The 3D cell reconstructions showed a notable decrease in fluorescence intensity after introducing VB_12_ to the CQDs-treated HeLa cells, suggesting that VB_12_ not only penetrated the cells but also interacted with CQDs inside the cells, leading to the quenching of CQD fluorescence. This observation demonstrated the significant potential of the synthesized CDs as optical nanoprobes for detecting VB_12_ within cells [176].

Liu et al. claimed that the selective and sensitive determination of Co^2+^ by the CDs would be promising for biological labeling, meeting the need for tracking the distribution of VB_12_ or other cobalt-containing medications within cells or organisms. They showed the bioimaging potential of the synthesized CDs in HeLa cells under in vitro conditions. The photoluminescent CDs, using inverted fluorescence microscopy, were monitored within the membrane and cytoplasmic regions of HeLa cells at λ_ex_ = 391 nm, indicating the CDs’ ability for efficient penetration of the cells. The authors assessed the impact of Co^2+^ on fluorescence cellular imaging when the cells were treated with 100 μM Co^2+^ and incubated for 20 min at room temperature. Results showed a meaningful quenching of cellular fluorescence, proposing that these CDs could be effectively used for bioimaging living cells at different doses of Co^2+^ and VB_12_ [183]. Nitrogen-doped CDs synthesized from ʟ-aspartic acid and 3,6-diaminoacridine hydrochloride demonstrated minimal cytotoxicity and excellent biocompatibility in in vitro studies conducted on HeLa cells using the MTT assay. The viability of HeLa cells decreased gradually as the concentration of N-doped CDs increased. However, the overall cell viability remained above 80% even at a high concentration of 500 μg/mL N-doped CDs. Upon incubating HeLa cells with 200 μg/mL N-doped CDs, they display a vivid blue fluorescence under 405 nm laser excitation in confocal laser scanning microscopy (CLSM). The fluorescence intensity of NCD-treated HeLa cells significantly decreased with 24 μM VB_12_ and was nearly extinguished at 60 μM VB_12_ concentration. This indicates that N-doped CDs could be effective fluorescent nanoprobes to detect VB_12_ within living cells [187].

Wang et al. also evaluated the bioimaging potential of fluorescent N,P co-doped CDs on HeLa cells. The MTT assay showed that cell viability could be maintained at 0.8 mg/mL N,P co-doped CDs. These nanoprobes were then introduced into HeLa cells via pipetting and incubated for 0.5 h to facilitate entry into the cells. Cells were subsequently washed with phosphate-buffered saline (PBS), and a remarkable alteration in cell morphology was observed using a laser confocal microscope. Subsequent addition of VB_12_ to the culture and a 1 h incubation followed by triple washing reduced background noise. Adding 500 μL of PBS to the culture medium led to a marked reduction in blue fluorescence, suggesting these CDs would be promising tools to be utilized in cell imaging [200]. Gao et al. also demonstrated that dual-emitting CPDs had a high ability in cell bioimaging. Bright blue fluorescence and yellow fluorescence were observed within the HeLa cells, indicating efficient entry of CPDs via endocytosis. In this setup, CPDs acted as the fluorescent probe, VB_12_ served as the recognition molecule, and the selective quenching of blue emission constituted the measurable output. The blue fluorescence of CPDs was significantly quenched upon the addition of VB_12_, while the yellow fluorescence showed only slight changes [210]. In another study, *Cannabis sativa*-derived N,S co-doped CDs exhibited high viability towards HeLa cells at a concentration range of 20–1,200 μg/mL, indicating very low toxicity and high biocompatibility. MTT results revealed that the cell viability could be kept over 90% at the maximum dose of 1,200 μg/mL. Images with a blue fluorescence signal were taken after incubating HeLa cells with these nanomaterials for 3 h using the CLSM at the λ_ex_ of 405 nm, affirming the internalization of N,S co-doped CDs via endocytosis, owing to their nanoscale size and hydrophilic characteristics [234].

He et al. assessed the biological potential of CDs by determining their cytotoxicity and potential for cell imaging. A low toxicity of CDs with only a slight reduction in cell activity (lower than 20%) was observed by increasing the CD concentration from 200 to 1000 μg/mL. The MDA-MB-231 human breast cancer cell line, initially non-fluorescent in bright field, exhibited intense orange fluorescence after being stimulated with a 488 nm laser following incubation with 600 μg/mL CDs. The overlay image in 15e (D) confirms the complete internalization of CDs by the cells, which also retained good morphological integrity. Since VB_12_ could quench fluorescence induced by these CDs, they could be potentially utilized for intracellular VB_12_ detection. Introducing various concentrations of VB_12_ to the cells led to a consistent decrease in cellular fluorescence intensity, while still enabling effective imaging. Therefore, CDs proved to be a viable fluorescent probe for VB_12_ detection within cells [178]. Yu et al. also assessed the toxicity of N-doped yellow fluorescent CDs towards MDA-MB-231 cells using the cell counting kit-8 (CCK-8) assay. After a 4 h co-culture with various N-doped CD concentrations, it was revealed that the survival rate of these cells exceeded 90% at a concentration of 20 mg/mL of N-doped CDs. Nonetheless, a gradual decrease in cell survival was observed at concentrations of more than 20 mg/mL. Accordingly, the authors selected a dose of 20 mg/mL of these CDs for subsequent cell imaging experiments. The results demonstrated that these nanomaterials were able to penetrate the cell nucleus over time, evidenced by increased yellow fluorescence after incubation [188].

Zhang et al. evaluated the potential of P,N co-doped CQDs as sensitive fluorescent nanoprobes for the detection and cellular imaging of VB_12_ and Co^2+^. The cytotoxicity results of P,N co-doped CQDs using MTT assays on PC12, human liver cancer (SMMC7721), human bronchial epithelial (BEAS-2B), and human lung cancer (A549) cell lines showed over 87% cell viability at an 800 μg/mL concentration of the nanomaterial. In this sensing design, P,N co-doped CQDs acted as the fluorescent probe, VB_12_/Co^2+^ functioned as the recognition molecules, and the fluorescence quenching in cytoplasm and nucleus provided the measurable signal. A significant decrease in fluorescence upon VB_12_ addition was found in SMMC7721 and BEAS-2B cells, indicating the P,N co-doped CQDs’ ability to detect VB_12_ in living cells. Besides, A549 and PC12 cells were utilized to monitor intracellular Co^2+^ levels, with fluorescence reduction observed shortly after Co^2+^ addition. In these experiments, P,N co-doped CQDs were not only distributed throughout the cytoplasm but also entered the nucleus of four different cell models [199]. Furthermore, Yu et al. reported blue fluorescence from *Saccharomyces*-derived N-doped CDs in A549 cells at λ_ex_ = 405 nm and pH 6.0, with a noticeable fluorescence reduction at higher pH levels. Comparative pH sensing in the human liver cell line LO2 and the human liver cancer cell line HepG2 cells revealed a decrease in the fluorescence intensity with rising pH across all tested cell types, confirming *Saccharomyces*-derived N-doped CDs as effective probes for intracellular pH monitoring. HepG2 cells displayed brighter fluorescence at pH 6.0 at λ_ex_ = 405 nm than LO2 cells [229], likely due to tumor cells’ slightly acidic microenvironment and enhanced phagocytosis [238]. This investigation explored the potential of specialized CDs in targeted biological sensing [229].

## Conclusions, Current Limitations, and Prospects

The exploration of optical biosensing technologies for VB_12_ detection has highlighted a significant shift from traditional analytical approaches toward more innovative, efficient, and sensitive methods. Central to this shift is the emergence of CD- and QD-based sensors, which have demonstrated unparalleled capabilities in terms of sensitivity, specificity, and the ability to operate in complex matrices. These advancements not only facilitate accurate and rapid VB_12_ quantification in food and pharmaceutical products but also extend to cutting-edge applications in cellular bioimaging and in vitro/in vivo monitoring, thus offering a multifaceted tool for biomedical research and diagnostics. The utility of CDs and QDs in optical biosensing is grounded in their unique photoluminescent properties, allowing for the detection of VB_12_ at nanomolar concentrations, far surpassing the capabilities of conventional methods. This sensitivity is crucial for early detection of VB_12_ deficiency, which can have profound health implications, including neurological disorders and anemia. Moreover, the ability of these nanomaterials to be functionalized for specific target recognition further enhances their specificity, ensuring that VB_12_ quantification is not confounded by the presence of similar biomolecules or interfering substances commonly found in biological samples. The integration of these nanosensors into cellular bioimaging represents a notable advancement, providing insights into the intracellular dynamics of VB_12_. This application not only furthers our understanding of VB_12_’s biological roles but also opens new avenues for investigating its therapeutic potentials and mechanisms of action within live cells. Furthermore, the exploration of non-invasive in vitro and in vivo analysis through these biosensors introduces the possibility of real-time monitoring of VB_12_ levels in organisms, a development that could revolutionize nutritional assessments and disease diagnostics.

Despite these promising advancements, the path toward widespread adoption of CD- and QD-based biosensors in clinical and nutritional science is fraught with challenges. The synthesis and functionalization of these nanomaterials often result in heterogeneity in sensor performance, posing significant hurdles to standardization and reproducibility. Additionally, the complexity of biological matrices in which VB_12_ detection is required demands sensors with improved robustness and selectivity, a goal that remains a work in progress. Moreover, concerns surrounding the biocompatibility and potential toxicity of these nanomaterials, especially for in vivo applications, necessitate further research to ensure their safe use. The translation of these advanced biosensing technologies into practical, user-friendly devices for point-of-care or at-home use also requires overcoming significant engineering, scalability, and cost barriers.

Looking ahead, the field of biosensing, particularly for VB_12_ detection using QDs and CDs, is ripe with opportunities for advancement and innovation. The continued exploration in advanced material engineering promises to refine the optical properties, stability, and biocompatibility of these nanomaterials, enhancing their efficacy as biosensors. The development of multiplexed and integrated sensing systems that can concurrently detect VB_12_ alongside other vital nutrients or biomarkers is another promising avenue, which could revolutionize comprehensive nutritional assessments and diagnostics. Moreover, the integration of these biosensors into smart platforms through digital technologies and the “Internet of Things” presents an exciting opportunity for real-time monitoring, data analysis, and personalized health management. Last, a focus on sustainable and green biosensing technologies emphasizes the importance of environmentally friendly materials and manufacturing processes, aligning with the principles of green chemistry and addressing environmental concerns associated with nanomaterials. Together, these future directions not only promise to overcome existing challenges but also unlock new possibilities in the realm of biosensing.

## Data Availability

Data sharing is not applicable as no new data was generated or analyzed in this study.
